# A New Subclade of *Leptosphaeria biglobosa* Identified from *Brassica rapa*

**DOI:** 10.3390/ijms20071668

**Published:** 2019-04-03

**Authors:** Zhongwei Zou, Xuehua Zhang, Paula Parks, Lindsey J. du Toit, Angela P. Van de Wouw, W. G. Dilantha Fernando

**Affiliations:** 1 Department of Plant Science, University of Manitoba, 66 Dafoe Road, Winnipeg, MB R3T 2N2, Canada; Zhongwei.Zou@umanitoba.ca (Z.Z.); xuehua.zhang@bayer.com (X.Z.); Paula.Parks@umanitoba.ca (P.P.); 2 Department of Plant Pathology, Washington State University Mount Vernon NWREC, Mount Vernon, WA 98273-4768, USA; dutoit@wsu.edu; 3 School of BioSciences, University of Melbourne, Parkville 3010, Victoria, Australia; apvdw2@unimelb.edu.au

**Keywords:** *Leptosphaeria maculans*, *Leptosphaeria biglobosa*, *Brassica napus*, *Brassica rapa*, *Brassica juncea*, *Brassica oleracea*, subclades, brassicae, canadensis, thlaspii, erysimii, australensis, occiaustralensis, ITS rDNA, actin, β-tubulin

## Abstract

Blackleg (Phoma stem canker) of crucifers is a globally important disease caused by the ascomycete species complex comprising of *Leptosphaeria maculans* and *Leptosphaeria biglobosa*. Six blackleg isolates recovered from *Brassica rapa* cv. Mizspoona in the Willamette Valley of Oregon were characterized as *L. biglobosa* based on standard pathogenicity tests and molecular phylogenetic analysis. These isolates were compared to 88 characterized *L. biglobosa* isolates from western Canada, 22 isolates from Australia, and 6 *L. maculans* isolates from Idaho, USA using maximum parsimony and distance analysis of phylogenetic trees generated from the ITS rDNA (internal transcribed spacer rDNA) sequence, and the *actin* and *β-tubulin* gene sequences. The *L. biglobosa* isolates derived from *B. rapa* collected in Oregon formed a separate subclade based on concatenated gene sequences or a single gene sequence, regardless of the analyses. Pathogenicity tests showed that these isolates failed to infect either resistant or susceptible *B. napus* cultivars, but caused severe symptoms on three *B. rapa* cultivars (Accession number: UM1113, UM1112, and UM1161), a *B. oleracea* var. *capitata* (cabbage) cultivar (Copenhagen Market), and two *B. juncea* cultivars (CBM, a common brown Mustard, and Forge). These findings demonstrated that the *L. biglobosa* isolates derived from a *B. rapa* crop in Oregon were genetically distinct from existing species of *L. biglobosa*, and constitute a new subclade, herein proposed as *L. biglobosa* ‘americensis’.

## 1. Introduction

*Leptosphaeria maculans* and *L. biglobosa* are two closely related fungal species that together form a species complex that causes blackleg or Phoma stem canker of crucifers, including *Brassica napus*, *B. juncea*, *B. oleracea*, and *B. rapa* [1,2]. *L. maculans* and *L. biglobosa* were previously described as virulent and weakly-virulent, respectively, with genetic differences and distinct phenotypic (disease) expression on oilseed rape (*B. napus*) leaves or stems [2]. During infection of *B. napus*, *L. maculans* produces large grey/green leaf lesions, and then grows down the vascular tissue to the stem, where the fungus causes necrotic stem cankers [2]. In contrast, *L. biglobosa* causes small, dark leaf lesions and typically is restricted to infection of the upper stem [3]. Consequently, *L. maculans* has been considered much more damaging than *L. biglobosa.*

*L. biglobosa* and *L. maculans* have been estimated to have diverged approximately 22 million years ago based on comparative genome sequencing analysis of 19 conserved proteins [4,5]. *L. maculans* has been categorized into two subclades: ‘Brassicae’ and ‘lepidii’; while *L. biglobosa* isolates have been categorized into six subclades: ‘Brassicae’, ‘canadensis’, ‘thlaspii’, ‘erysimii’, ‘australensis’, and ‘occiaustralensis’ [6,7]. *L. biglobosa* ‘brassicae’ is the most common of the *L. biglobosa* species and has been found in most oilseed rape growing regions [8]. Although isolates of *L. biglobosa* are often found in association with *L. maculans*, *L. biglobosa* ‘brassicae’ is the sole subspecies that has been identified in China to date [8]. *L. biglobosa* ‘canadensis’ has been isolated from both *B. napus* and *B. juncea* crops in both Canada and Australia, and is most closely related to *L. biglobosa* ‘brassicae’ [9,10]. The remaining four *L. biglobosa* subspecies are less common and have only been reported in specific situations: *L. biglobosa* ‘thlaspii’ was obtained from the cruciferous weed, *Thlaspi arvense*, in central Canada; *L. biglobosa* ‘erysimii’ was obtained from a weed of an *Erysimum* sp., also in Canada [6]; *L. biglobosa* ‘australensis’ has been isolated from *B. napus* and *B. juncea* in the United States of America and Australia [11,12]; and *L. biglobosa* ‘occiaustralensis’ has been isolated mainly from oilseed rape cultivars with resistance derived from *B. rapa* sp. *sylvestris*, and wild radish (*Raphanus raphanistrum*) in western Australia, Chile, and Georgia [7,9]. *L. maculans*–*L. biglobosa* species complex had been separated into seven distinct groups using the sequence of the ITS region. In these studies, ITS rDNA, and *actin* and *β-tubulin* sequences have been widely used for the phylogeny analysis of the *Leptosphaeria* complex and new subclade identification. Parsimony and distance analyses separated these species with groups corresponding to specific host plants and geographic origin [6]. In 2008, Vincenot et al. identified a new subclade of *L. biglobosa* (*L. biglobosa* ‘occiaustralensis’) using ITS rDNA, *actin*, and *β-tubulin* sequences in western Australia [7]. The phylogeny of *L. biglobosa* isolates on the American continent has also been analyzed by using ITS rDNA, *actin*, and *β-tubulin* sequences [9]. In addition, other important host-pathogen systems, such as fusarium and wheat, where *F. graminearum* causes the fusarium head blight (FHB) disease, conserved gene sequences, i.e., ITS, histone H3, elongation factor 1-α, and *β-tubulin*, have been widely used to identify species of *Fusarium* [13,14]. More recently, 16 monophyletic species have been identified within the *Fusarium graminearum* species complex using a high-throughput multilocus assay of portions of housekeeping genes [15,16,17]. *L. maculans* has spread recently into many areas where only *L. biglobosa* was present, suggesting that *L. biglobosa* may have evolved earlier than *L. maculans* from a common ancestor [18]. 

In 2014, a vegetable seed grower in the Willamette Valley in Oregon, USA submitted samples from a certified organic seed crop of the *B. rapa* vegetable cv. Mizspoona. The plants had typical foliar and stem symptoms of blackleg. Six isolates of *Leptosphaeria* were recovered from the *Brassica rapa* seed crop, Mizspoona, and were characterized using standard pathogenicity tests and sequence analysis of the ITS rDNA and *actin* and *β-tubulin* genes to determine the species of these isolates. 

## 2. Results

### 2.1. Pathogenicity Tests 

**Figure 1:**
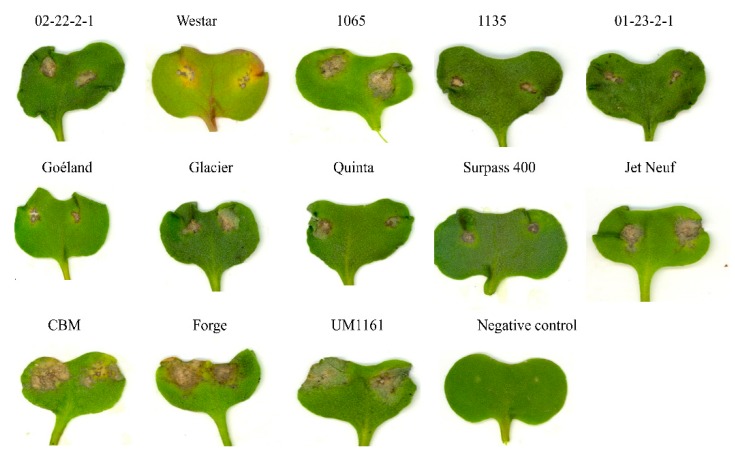
Disease symptoms expressed 14 days’ post-inoculation (dpi) of cotyledons of *Brassica napus* differential cultivars/lines 02-22-2-1, Westar, 1065, 1135, 01-23-2-1, Goéland, Glacier, Quinta, Surpass 400, and Jet Neuf; two *B. juncea* lines, a common brown mustard (CBM) and Forge; and one *B. rapa* line, UM1161 (see Table S2 for details of cultivars/lines), with isolate Phl004 of *Leptosphaeria biglobosa* obtained from the Willamette Valley of Oregon (see Table S1 for details of isolates). Seedlings of the *B. napus* cv., Westar, inoculated with water were used for the negative treatment. Small, dark brown, necrotic lesions without pycnidia were observed on cotyledons of the *B. napus* differential cultivars/lines.

Small, dark brown, necrotic lesions without pycnidia were observed on cotyledons of the *B. napus* differential cultivars or lines, including the universal susceptible cultivar, Westar, when inoculated with each of the six isolates (Phl002 (*Phoma lingam*002) to Phl007) of *Leptosphaeria* derived from a *B. rapa* vegetable seed crop in the Willamette Valley of Oregon (Figure 1). Conversely, all six isolates caused susceptible reactions on the *B. juncea* cultivars, Forge and CBM; and on the three *B. rapa* lines, UM1161, UM1113, and UM1112, by 11 dpi (Figure 1 and Table 1). Six additional isolates (Phl010 to Phl015) derived from *B. napus* infected plants collected from Idaho (Table S1) were all virulent on Westar, as expected, and showed avirulent or virulent reactions on a range of cultivars of *B. napus* differing in resistance genotypes. There were no disease symptoms or a hypersensitive reaction on the *Brassica* germplasm inoculated with water.

Since all the *B. rapa*-derived isolates were avirulent on all the *B. napus* cultivars and lines tested, but virulent on several *B. juncea* (CBM and Forge) and *B. rapa* (UM1113, UM1112, and UM1161) cultivars and lines, it was deemed necessary to assess whether these isolates were *L. maculans* or *L. biglobosa*. Therefore, the *B. rapa*-derived isolates from Oregon were subjected to a PCR (Polymerase chain reaction) assay of the ITS rDNA, and the amplified sequences used for species identification.

### 2.2. Characterization of B. rapa-Derived Isolates from the Willamette Valley of Oregon 

**Figure 2:**
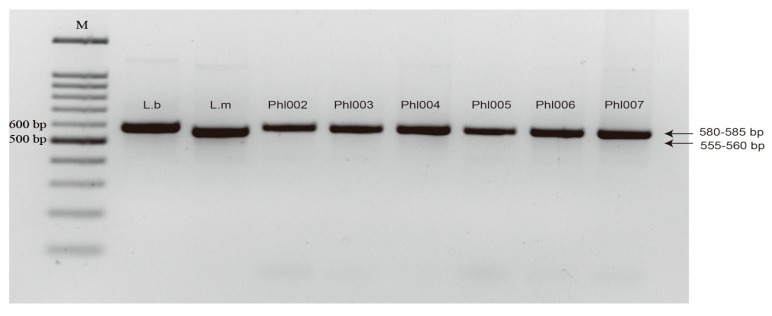
PCR identification of a *Leptosphaeria* complex with the primers, PN3 and PN10, using genomic DNA of isolates Phl002 to Phl007 obtained from an organic vegetable *Brassica rapa* seed crop of the cv., Mizspoona, grown in the Willamette Valley of Oregon. Lb. and Lm. were reference isolates for the 555 to 560 bp and 580 to 585 bp amplified bands of *L. biglobosa* and *L. maculans* isolates, respectively. M, 100 bp DNA ladder.

PCR amplification of the six Oregon isolates of *Leptosphaeria* obtained from a *B. rapa* seed crop, using the PN3 and PN10 primers, illustrated that each of the isolates, Phl002 to Phl007, produced amplified DNA bands matching the size of the *L. biglobosa* DNA band (Figure 2). Evaluation of the mating type alleles indicated that none of the six isolates could be positively amplified and characterized to either of the two known mating types of *L. maculans*, i.e., the isolates could not be categorized as *L. maculans*, which supported their possible assignment to *L. biglobosa* based on the ITS rDNA size. 

The ITS rDNA sequences and the *actin* and *β-tubulin* gene sequences were obtained from *B. rapa*-derived isolates, Phl002 to Phl007, and compared with sequences deposited in the NCBI (National Center for Biotechnology Information) database. The *B. rapa*-derived isolates (Phl002 to Phl007) had 99.00% or 99.01% ITS rDNA sequence similarities (deposited in Genbank: MG321243) to the ITS rDNA sequence from *L. biglobosa* ‘brassicae’ DQ133890, compared with only 95.59% to 96.01% for that of *L. maculans* JX648199. Similarly, the *actin* gene sequences (deposited in Genbank: MG282088) of the *B. rapa*-derived isolates showed 99.00% to 99.02% and 93.56% to 93.58% similarities to *actin* sequences of the *L. biglobosa* ‘brassicae’ group strain (AY748949) and *L. maculans* ‘brassicae’ group strain (AY748971), respectively. In addition, the *B. rapa*-derived isolates had 99.00% to 99.02% *β-tubulin* gene (deposited in Genbank: MG282089) similarities to the *L. biglobosa* ‘canadensis’ group strain, AY749006 compared with only 92.45% or 92.46% for that of *L. maculans* ‘brassicae’ group strain AY749018, in the NCBI database (Table S3). In summary, the six *B. rapa*-derived isolates from Oregon were categorized as *L. biglobosa* through sequencing of the ITS rDNA and the *actin* and *β-tubulin* genes, and based on pathogenicity tests.

### 2.3. Identification of a New Subclade of L. biglobosa by Phylogenetic Analysis 

All 88 isolates from western Canada were characterized as *L. biglobosa* ‘canadensis’, while 12 isolates from Australia had been characterized as *L. biglobosa* ‘canadensis’ and six as *L. biglobosa* ‘occiaustralensis’. Since 88 isolates from western Canada and 12 isolates from Australia were characterized as the same subspecies, *L. biglobosa* ‘canadensis’, 9 and 6 isolates were randomly selected from Canada and Australia, respectively, for phylogenetic analysis. Deposited sequences of ITS rDNA, *actin*, and *β-tubulin* in Genbank of *L. biglobosa* subspecies were extracted as references in the analysis (Table S1). The nucleotide identity between different subclades of *L. biglobosa* isolates of ITS rDNA sequences ranged from 91.33% to 99.64%. Only two nucleotide differences were detected between *B. rapa* derived isolate (i.e., an Oregon isolate) and *L. biglobosa* ‘brassicae’, and they shared the most sequence similarity (99.64%) among the subclades of *L. biglobosa*. A total of 9, 6, 18, 26, and 28 SNPs (single nucleotide polymorphism)/Indels were identified between the *B. rapa* derived isolate and *L. biglobosa* ‘canadensis’, ‘occiaustralensis’, ‘australensis’, ‘erysimii’, and ‘thlaspii’, respectively (Figure S1). The nucleotide identity of the *actin* gene sequences between different subclades of *L. biglobosa* isolates ranged from 94.34% to 99.13%. Similar with ITS rDNA, the *actin* sequence of *B. rapa* derived isolate showed the highest identity with *L. biglobosa* ‘brassicae’ with only three SNPs detected. In total, 9, 10, 14, 17, and 23 SNPs/Indels were filtered in the *actin* sequence of *B. rapa* derived isolate when compared to *L. biglobosa* ‘canadensis’, ‘occiaustralensis’, ‘australensis’, ‘erysimii’, and ‘thlaspii’, respectively (Figure S2). While the ITS rDNA and *actin* sequences were most similar with those of *L. biglobosa* ‘brassicae’, the *β-tubulin* gene sequences of *B. rapa* derived isolates showed the greatest identity with *L. biglobosa* ‘canadensis’ and contained four SNPs. The nucleotide acid identity of the *β-tubulin* gene sequences between different subclades of *L. biglobosa* isolates ranged from 92.77% to 98.72%. In addition, there were 13, 12, 17, 21, and 31 SNPs/Indels in *β-tubulin* between *B. rapa* derived isolate and *L. biglobosa* ‘brassicae’, ‘occiaustralensis’, ‘australensis’, ‘erysimii’, and ‘thlaspii’, respectively (Figure S3).

**Figure 3:**
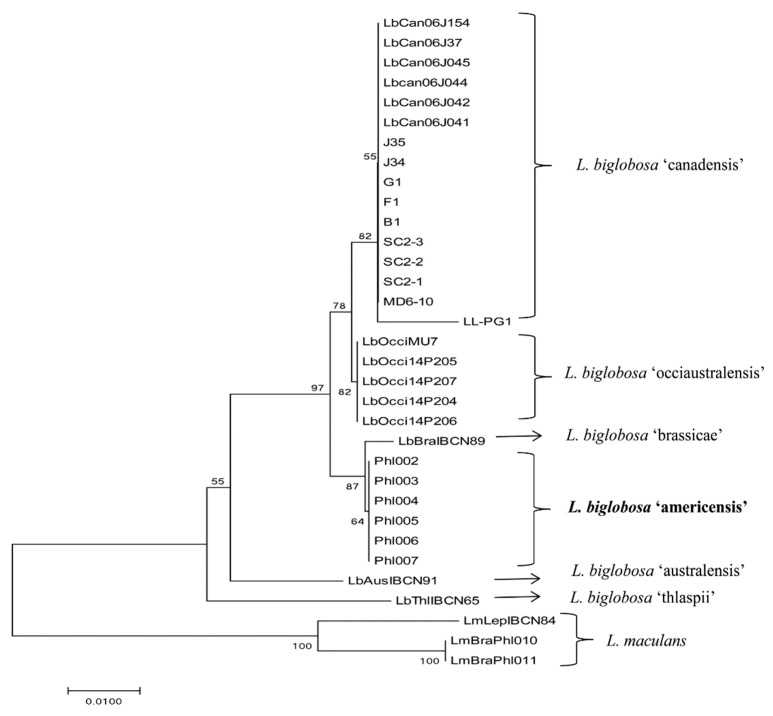
Phylogenetic analysis of the *Leptosphaeria maculans*–*L. biglobosa* species complex based on maximum parsimony analysis of the concatenated sequences of the internal transcribed spacer (ITS) region of ribosomal DNA, *actin*, and *β-tubulin* (one of two parsimony trees). Bootstrap values (1000 replications) are indicated as a percentage above the node. The length of the tree is 449 steps (CI = 0.84, RI = 0.91). The tree was similar to trees constructed by neighbor-joining (NJ) or maximum likelihood (ML) analyses. Three *L. maculans* isolates were included as outgroup control isolates.

Alignment of the ITS rDNA sequences resulted in a dataset of 573 characters of which 43 (7.5%) were considered to be informative characters. The *actin* sequence alignment resulted in a dataset of 477 characters of which 43 (9.0%) were parsimony informative. The *β-tubulin* sequence alignment resulted in 501 characters of which 26 (2.6%) were parsimony informative. Finally, a set of data with concatenate sequences of these three conserved genes resulted in a dataset of 1551 characters of which 112 (7.2%) were parsimony informative for phylogenetic tree construction (CI (Consistency index) = 0.84, RI (Retention index) = 0.91). A total of six subclades contained all the *L. biglobosa* isolates by concatenated ITS rDNA, *actin*, and *β-tubulin* sequences, which were differentiated clearly from the two *L. maculans* subclades (Figure 3). As expected, the isolates, ICBN89 and IBCN91, grouped in the *L. biglobosa* ‘brassicae’ and *L. biglobosa* ‘australensis’ clades, respectively. The *L. biglobosa* ‘thlaspii’ isolates were differentiated from a strongly supported clade that included all other subclades, and was most closely aligned with the *L. maculans* subclades. *L. biglobosa* ‘australensis’ and *L. biglobosa* ‘erysimii’ isolates formed one branch based on the ITS rDNA sequence (Figure S4). The close phylogenetic relationship observed between *L. biglobosa* ‘canadensis’ and *L. bilobosa* ‘occiaustralensis’ was similar to that published by Vincenot et al. (2008) [7]. Interestingly, the six *B. rapa*-derived *L. biglobosa* isolates from Oregon formed a distinct subclade most closely related to *L. biglobosa* ‘brassicae’ isolates, and were clearly separated from the outgroup of *L. maculans* isolates (Figure 3). The distance and parsimony analyses on a concatenated set of three gene sequences resulted in threes of similar topology to those analyses on a single sequence of ITS rDNA or the *actin* gene (Figures S4 and S5). Phylogenetic trees constructed using the maximum likelihood, minimum evolution, and unweighted pair group method with arithmetic mean (UPGMA) were very similar to those obtained using the neighbour joining and parsimony analyses, and showed that the *B. rapa*-derived isolates, Phl002 to Phl007, clustered into a unique subclade distinct from other *L. biglobosa* isolates and from *L. maculans* isolates (data not shown). 

The six *B. rapa*-derived isolates formed a distinct subclade using the *β-tubulin* gene sequences. While the ITS rDNA and *actin* sequences placed the *B. rapa*-derived isolates closest to the *L. biglobosa* ‘brassicae’ subclade, the *β-tubulin* gene sequences of the *B. rapa*-derived isolates showed the greatest identity with *L. biglobosa* ‘canadensis’ isolates (Figure S6). This probably reflected the greater level of sequence polymorphism detected in the *β-tubulin* sequences between *B. rapa*-derived isolates and *L. biglobosa* ‘brassicae’ isolates compared to those of the *L. biglobosa* ‘canadensis’ isolates (Figure S3). Since the *B. rapa*-derived isolates fell into a distinct clade, based on the conserved gene regions and concatenated sequences analyzed, a new subclade, *L. biglobosa* ‘americensis’, is proposed.

### 2.4. Differences in the Pathogenicity of Isolates of L. biglobosa ‘americensis’ and Other L. biglobosa Subspecies

**Figure 4:**
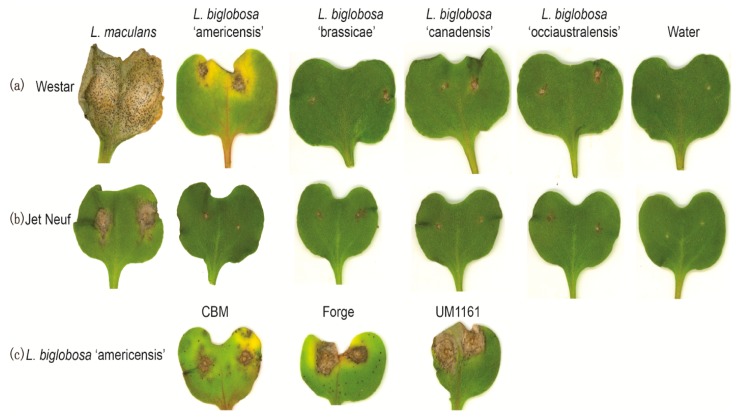
Disease symptoms on cotyledons of *Brassica napus* cvs Westar (**a**) and Jet Neuf (**b**) 14 days’ post inoculation (dpi) with isolates of *L. maculans* (06LM), *Leptosphaeria biglobosa* ‘americensis’ (isolate Phl004), *L. biglobosa* ‘brassicae’ (LL1-PG1), *L. biglobosa* ‘canadensis’ (06J37), *L. biglobosa* ‘occiaustralensis’ (14P207), and water. (**c**) Disease symptoms on cotyledons of *Brassica juncea* cultivar. Common brown mustard (CBM), *B. juncea* cv. Forge, and *B. rapa* line UM1161 14 dpi with *L. biglobosa* ‘americensis’ (Phl004).

**Figure 5:**
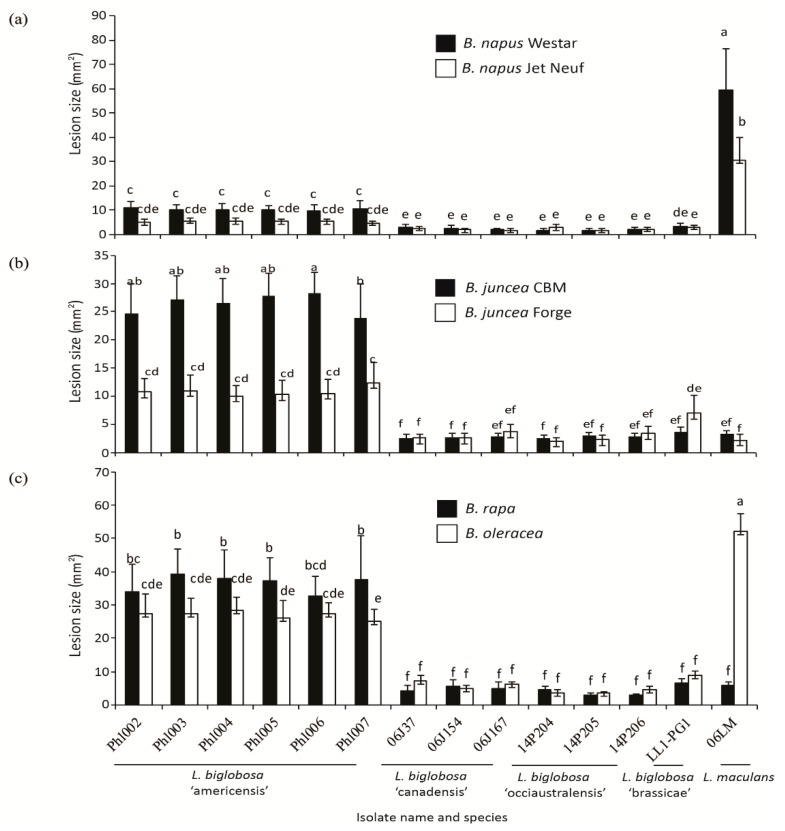
Pathogenicity assessment of 14 *Leptosphaeria* isolates belonging to four subclades of *L. biglobosa* and one isolate of *L. maculans* as a control treatment: *L. biglobosa* ‘americensis’ isolates (Phl002 to Phl007); *L. biglobosa* ‘canadensis’ isolates (06J37, 06J154, 06J167); *L. biglobosa* ‘occiaustralensis’ isolates (14P207, 14P205, 14P206); *L. biglobosa* ‘brassicae’ isolate (LL1-PG1); and *L. maculans* isolate (06LM). Each isolate was inoculated onto seedlings of each of two *Brassica napus* cvs, Westar and Jet Neuf (**a**); two *B. juncea* cvs., Forge and common brown mustard (CBM) (**b**); *B. rapa* turnip cv., Mizspoona, from the Willamette Valley of Oregon; and *B. oleracea* cabbage cv., Copenhagen Market (**c**). The lesion sizes were quantified 14 days’ post-inoculation (dpi) using Assess 2.0 (American Phytopathological Society, St Paul, MN) for each test, isolate, and *Brassica* cultivar as described in the main text, and statistical analyses performed using SAS Version 9.4 (SAS Institute, Inc., Cary, NC) for analysis of variance (ANOVA) and means comparison with Tukey’s Honestly Significant Difference Studentized range test at *p* ≤ 0.05. Each bar represents the mean and standard deviation of the lesion size for a particular *Brassica* species. Different letters (from ‘a’ to ‘f’) over the bars indicate the significant differences (*p* < 0.05).

To determine whether isolates of this proposed new subspecies, *L. biglobosa* ‘americensis’, have similar disease profiles to isolates of the other *L. biglobosa* species, pathogenicity tests were carried out with representative isolates from *L. biglobosa* ‘canadensis, *L. biglobosa* ‘brassicae’, and *L. biglobosa* ‘occiaustralensis’. Compared to the severity of cotyledon symptoms caused by isolates of the other subclades of *L. biglobosa*, the *L. biglobosa* ‘americensis’ isolates caused significantly larger lesions on cotyledons of the cultivar, Westar (Figure 4 and Figure 5a), although these lesions were still significantly smaller than the lesions caused by the *L. maculans* control isolate. Alternatively, on the cultivar, Jet Neuf, no significant differences in lesion size were detected among the subclades of the *L. biglobosa* isolates (Figure 4 and Figure 5a). In the pathogenicity test of the two *B. juncea* cultivars (CBM and Forge), lesion sizes on CBM inoculated with *L. biglobosa* ‘americensis’ isolates were all significantly larger than those on plants inoculated with the other *L. biglobosa* isolates, which were all rated in the susceptible or intermediate categories (Figure 5b). Most of the *L. biglobosa* ‘americensis’ isolates caused significantly larger lesions on Forge seedlings compared to lesions caused by isolates of *L. biglobosa* ‘canadensis’ and ‘occiaustralensis’, but not of ‘brassicae’ (Figure 5b and Figure S7). The lesion sizes were significantly larger on *B. rapa* (cultivar ‘Mizspoona’, where *L. biglobosa* ‘americensis’ isolates identified from) and *B. oleracea* plants inoculated with the *L. biglobosa* ‘americensis’ isolates than on plants inoculated with isolates of the other subclades of *L. biglobosa* (Figure 5c). These results suggest that the *L. biglobosa* ‘americensis’ isolates have a significantly different disease profile to the other *L. biglobosa* subspecies tested. 

## 3. Discussion

This study describes isolates of a proposed new subclade of *L. biglobosa* obtained from a certified organic *B. rapa* vegetable seed crop of the cv., Mizspoona, grown in the Willamette Valley of Oregon, USA in 2013 to 2014 that had developed classic symptoms of blackleg. Pathogenicity and molecular characterization clearly identified these isolates as belonging to a distinct subclade compared to isolates of the six known subclades of this species. We propose naming the new subclade *L. biglobosa* ‘americensis’. 

Relationships between members of the two *Leptosphaeria* species, *L. maculans* and *L. biglobosa*, based on three conserved DNA regions yielded fairly consistent phylogenetic trees and were extremely comparable to previously published studies [6,7,11]. For example, previous studies found that isolates of the subclade, *L. biglobosa* ‘occiaustralensis’, were most closely related to isolates of *L. biglobosa* ‘canadensis’, while isolates of *L. biglobosa ‘*australensis’ and ‘erysimii’ were closely related to each other [6,7]. Indeed, the topologies of these subclades are consistent with the data generated in this study. In addition, *L. biglobosa* ‘thlaspii’ isolates were most closely related to *L. maculans* isolates in the phylogenetic trees constructed previously and in this study. The distance and parsimony analyses on concatenated sequences or a single gene sequence with high bootstrap value supported that subclades including the six *L. biglobosa* isolates is indeed a new subspecies. *L. biglobosa* ‘canadensis’ was close to *L. biglobosa* ‘occiaustralensis’ through concatenated/single gene sequence analysis. The six *B. rapa* derived isolates formed an independent group and was mostly close to *L. biglobosa* ‘brassicae’ when using concatenated sequences of three conserved genes and consistent with those trees constructed by single sequence of ITS rDNA or *actin*. The inconsistent tree obtained by phylogeny analysis using the *β-tubulin* sequence, which showed the new six isolates as closer to *L. biglobosa* ‘canadensis’, reinforced that these six isolates cannot be categorized into either ‘brassicae’ or ‘canadensis’ and formed a distinct group. To eliminate the bias of the phylogenetic tree constructed by the single gene sequence, concatenated sequences of three conserved genes were applied for parsimony analyses, the result being that these six isolates formed a separate subclade that was closer to *L. biglobosa* ‘brassicae’. More genomic or transcription data will provide further insights on the characterization of the new subclade in the future for investigation of the difference between this new subclade and other subclades of *L. biglobosa*. Trees obtained through parsimony analyses were similar to those obtained by the NJ method, and sufficient bootstraps supported the hypothesis that the *B. rapa*-derived isolates formed a monophyletic clade. Phylogenic analysis using NJ, parsimony, maximum likelihood, and minimum evolution analyses resulted in trees of similar topologies, with greater bootstrap values for the NJ trees compared with the other trees. It remains unclear why the different subspecies of *L. biglobosa* are so distinct from one another. With the exception of *L. biglobosa* ‘brassicae’ and *L. biglobosa* ‘canadensis’, generally, few isolates have been cultured from each of the subspecies, making population surveys difficult. Therefore, limited information is known regarding gene flow across geographical regions. 

Isolates of *L. biglobosa*, which can coexist on host plants with isolates of *L. maculans*, typically are associated with upper stem lesions on infected *Brassica* plants, and are considered weakly virulent or even avirulent. *L. biglobosa* isolates have only caused significant losses in areas with high summer temperatures, e.g., in Poland [19]. Vincenot et al. (2008) demonstrated that *L. biglobosa* ‘occiaustralensis’ isolates were more virulent than *L. biglobosa* ‘canadensis’ or ‘australensis’ isolates, and *L. biglobosa* ‘brassicae’ isolates produced large leaf lesions on *B. napus* plants [7]. The pathogenicity of *L. biglobosa* ‘americensis’ isolates on *Brassica* spp. in this study indicated that these isolates were intermediate in virulence on *B. napus* and caused more severe disease on *B. juncea* and *B. rapa*. Van de Wouw et al. (2008) reported that *L. biglobosa* ‘canadensis’ isolates induced similar size lesions on *B. juncea* cotyledons as *L. maculans* isolates [10]. In contrast, *L. biglobosa* ‘americensis’ isolates in this study caused significantly larger lesions than *L. biglobosa* ‘canadensis’ isolates on both resistant and susceptible lines of *B. napus*, and large lesions on *B. juncea* and *B. rapa* lines. These results were confirmed by the accumulation of lignified material observed in stained, infected cotyledons, with more diffuse and less lignified material in the cotyledons caused by *L. biglobosa* ‘americensis’ and *L. maculans* isolates than isolates of other subclades of *L. biglobosa*. It remains unclear why the different subspecies of *L. biglobosa* show different reactions on different *Brassica* species, however, it raises the question of whether effector genes are present in these *L. biglobosa* subspecies and whether they may play a role in the pathogenicity of the various *Brassica* species. The genome sequencing of a subset of *L. biglobosa* species has shown different suites of effector-like proteins, thus supporting this hypothesis, however, the functions of these effector-like proteins remain unknown in these subspecies [4]. 

In contrast to isolates of other *L. biglobosa* subclades, *L. biglobosa* ‘americensis’ isolates characterized in this study represent the first *L. biglobosa* subclade derived from *B. rapa* that caused severe symptoms on *B. rapa*, *B. oleracea*, and *B. juncea* cultivars. The evolution of *Leptosphaeria* isolates appears to be influenced by internal and external forces, including mutation, reproduction, gene flow, genetic drift, natural selection (e.g., temperature and geography) [20], and host specificity [21]. Unlike the well understood gene-for-gene relationship of *R* genes and corresponding avirulence genes in the *B. napus*–*L. maculans* pathosystem [22], it remains unclear whether a gene-for-gene interaction exists among *L. biglobosa* isolates and *Brassica* species. The results of this study validated and enriched the known diversity of *L. biglobosa* isolates, and provide a resource not only to help determine whether the evolutionary process of *L. biglobosa* isolates is influenced by geographic location and host selection pressures, but also to underline the possible existence of plant defense responses among *Brassica* species and *L. biglobosa* isolates. As reflected by the more severe disease symptoms associated with the *L. biglobosa* ‘americensis’ isolates on cultivars of three *Brassica* spp. evaluated in this study compared to isolates of other subclades, further research is needed to investigate potential differences in resistance to *L. biglobosa* ‘americensis’ isolates vs. isolates of other subclades, and to find sources of resistance to isolates of the different subclades of *L. biglobosa*.

## 4. Materials and Methods 

### 4.1. Isolate Collection 

A total of 122 *Leptosphaeria* isolates (12 from the USA, 88 from western Canada, and 22 from Australia) were included in this study (Table S1). For the USA isolates, six were cultured from stems and roots of canola crop residues from northern Idaho (Phl010 to Phl015), and six were cultured from *B. rapa* stems of six plants of the cv., Mizspoona, sampled from a certified organic seed crop grown in the Willamette Valley, Oregon (Phl002 to Phl007) (Table S1). Of the 88 isolates from western Canada, 67 were sampled from infected stems or stubble sections of canola crops (*B. napus*) grown in 2010, 2011, 2012, 2013, or 2014. The remaining 21 isolates were sampled from the dockage of canola (*B. napus*) seeds. All Canadian and USA isolates were cultured by plating small pieces of surface-sterilized stubble, stem, or seed onto potato dextrose agar (PDA) medium amended with chloramphenicol (100 mg/L medium) (for USA isolates) or V8 agar amended with 0.35% streptomycin sulfate (for Canadian isolates) (Table S1). The individual isolates were then obtained by streaking pink cirrhi that developed from pycnidia on the infected tissues, followed by hyphal tip transfers from single colonies that germinated from the pycnidiospores separated on the agar surface. The isolates were then subjected to a second round of single-spore isolation. The 22 Australian isolates were collected from infected canola stems in field trials at the end of the 2006 and 2014 growing season. The stems were left to mature over the summer, and once pseduothecia (sexual fruiting bodies) formed the following year, ascospores were obtained as described previously [23]. In summary, to obtain single-spore cultures, individual ascospores were collected using a dissecting microscope with a sterilized needle. *L. maculans* ‘brassicae’ isolate (06LM) collected from Canada was used as a control isolate throughout this study, and is hereafter referred to as *L. maculans*. All isolates were maintained on V8 agar medium. 

### 4.2. Initial Pathogenicity Testing of the Oregon B. rapa-Derived Leptosphaeria Isolates on Brassica Species 

Pycnidiospore inoculum of each isolate of *Leptosphaeria* was harvested by flooding 8- to 11-day-old cultures of the appropriate single-spore isolate using distilled water (2 mL/plate). The concentration of spores was adjusted to 2 × 10^7^ spores/mL for the cotyledon inoculation test. The seed of 10 *B. napus* differential cultivars or lines carrying different *L. maculans* related resistance genes, eight *B. juncea* cultivars or lines, and eight *B. rapa* cultivars or lines (Table S2) were each sown into 96-cell flats filled with Pro-Mix BX (Premier Tech, Rivière-du-Loup, QC, Canada) and placed in a growth chamber at 16 °C (night) and 21 °C (day) with a 16-h photoperiod/day. The cotyledons of each 7-day-old seedling were each punctured and inoculated with a 10 µL droplet of inoculum at each of two wound sites/cotyledon (four wound sites/seedling) as previously described [24]. As a negative control treatment, the cotyledons of seedlings of each cultivar of each *Brassica* species were punctured and inoculated with a droplet of water instead of a spore suspension. A rating scale of 0 to 9, based on lesion size, chlorosis or necrosis, and the presence of pycnidia, was used to evaluate the interaction phenotype 11 to 14 dpi as described by Zhang et al. (2016) [24]. For each isolate screened, an average score was calculated from the 24 inoculation sites (four wound sites/plant for each of six plants): A mean of 6.1 to 9.0 was considered a susceptible (S) reaction, 4.6 to 6.0 an intermediate (I) reaction, and ≤4.5 a resistant (R) reaction [24,25]. 

### 4.3. PCR Identification of Isolates 

A mixture of hyphae, pycnidia, and pycnidiospores was collected from a V8 agar plate of each isolate of *Leptosphaeria*, and stored in 1.5 mL micro-centrifuge tubes at −20 °C before DNA extraction. DNA extraction was performed using the cetyl trimethylammonium bromide (CTAB) method described previously by Calderon et al. (2002) with some minor modifications [26].

To confirm the species of the *B. rapa*-derived isolates from Oregon and six isolates from *B. napus* infected tissues in Idaho, the ITS rDNA was amplified using ITS F (PN3) and ITS R (PN10) primers designed from the 18S rDNA and 28S rDNA of *Saccharomyces cerivisiae*, respectively (Table 2) [6]. This primer set generates a 555 to 560 bp fragment for *L. maculans* and a 580 to 588 bp fragment for *L. biglobosa* [6]. A multiplex PCR assay developed by Cozijnsen and Howlett (2003) was employed to characterize the mating type of those isolates [27]. Therefore, we were able to further clarify whether these isolates can be categorized as *L. maculans* or not. A 686 bp band was amplified from all *MAT1-1* isolates, while a 443 bp band was amplified from all *MAT1-2* isolates (Table 2) [27]. The annealing temperatures for primers used in this study are presented in Table 2. 

### 4.4. Conserved Gene Sequencing and Sequence Alignment

For sequencing the ITS rDNA, *actin* gene, and *β-tubulin* gene of each of the 88 isolates from western Canada and 12 isolates from USA, PCR amplification was performed as described above. The ITS rDNA, *actin*, and *β-tubulin* PCR products were then sent to AGTC (Advanced Genetic Technologies Center, University of Kentucky, Lexington, KY) for sequencing in both directions. Of the total 122 isolates, determined and available ITS rDNA, *actin* gene*,* and *β-tubulin* gene sequences of 22 isolates from Australia were included as different subspecies of *L. biglobosa*. In addition, 28 previously published sequences of these conserved genes available in GenBank were also employed as references for the analysis (Table S1). For the conserved gene similarity analysis, an *L. biglobosa* (Table S3) DNA sequence similarity analysis was performed using the BLAST search tool [28].

### 4.5. Phylogenetic Analysis

Initially, sequence alignments were carried out on the ITS rDNA, *actin*, and *β-tubulin* sequences from the six *B. rapa*-derived isolates compared to ITS rDNA, *actin*, and *β-tubulin* sequences from *L. biglobosa* isolates recovered from different *Brassica* spp. (Table S1) using Clustal Omega [29] with default parameters (https://www.ebi.ac.uk/Tools/msa/clustalo/) and Genedoc [30]. Since the Canadian isolates were characterized to be *L. biglobosa* ‘canadensis’ and to eliminate the genetic bias of using gene sequences of the same isolates in the phylogenetic analysis, the sequences from the six Oregon isolates were compared to randomly selected and representative isolates of the *L. biglobosa*-*L. maculans* complex using ITS rDNA (31 determined sequences and 8 deposited sequences from Genbank), *actin* (24 determined sequences and 7 deposited sequences from Genebank), and *β-tubulin* (20 determined sequences and 6 deposited sequences from Genbank) (Table S1). Therefore, we were able to determine whether the six *B. rapa*-derived isolates formed a separate and consistent species clade compared to previously characterized *L. biglobosa* subspecies. Analyses of sequences and phylogenetic relationships were calculated using MEGA6 [31]. The phylogenetic tree was reconstructed using the neighbor-joining method by the Kimura two-parameter model, with bootstrap calculation using 1000 replications. Phylogenetic analyses were also conducted on the conserved gene sequences using maximum parsimony (MP) analysis with the subtree-pruning-regrafting algorithm and maximum likelihood (ML) with the Kimura two-parameter distance method using MEGA6 [31]. For each analysis, 1000 bootstrap resamplings were used to assess clade stability, and *L. maculans* isolates were designated as the outgroup in all rooted trees. 

### 4.6. Pathogenicity of L. biglobosa Subclade Isolates on Brassica Species 

The *B. rapa*-derived isolate Phl004, *L. biglobosa* ‘brassicae’ isolate LL1-PG1, *L. biglobosa* ‘canadensis’ isolate 06J37, and *L. biglobosa* ‘occiaustralensis’ isolate 14P207 were inoculated onto punctured cotyledons of seedlings of *B. napus* cultivars, Westar and Jet Neuf, seven days after seeding, as described above. In addition to the pathogenicity tests done on two *B. napus* cultivars (Westar and Jet Neuf), the severity of symptoms caused by isolates of each subclade of *L. biglobosa* was evaluated on two *B. juncea* cultivars, CBM and Forge, a *B. rapa* line (a turnip from the Willamette Valley), and the *B. oleracea* cabbage cultivar, Copenhagen Market. Cotyledons of seedlings inoculated with *L. maculans* isolate 06LM and with water served as positive and negative control treatments, respectively, for each of the *Brassica* species tested. Each isolate was inoculated onto the cotyledons of at least six plants, and the experiment was repeated two or more times. Lesion size was quantified 14 dpi using Assess 2.0 (American Phytopathological Society, St Paul, MN, USA) for each experiment, isolate, and *Brassica* cultivar interaction, as described above, and statistical analyses performed using SAS Version 9.4 for ANOVA and means comparison with Tukey’s HSD Studentized range test at *p* ≤ 0.05. 

## Figures and Tables

**Table 1:** Mean pathogenicity scores and their inferred disease resistance for seedlings of the *Brassica* species in response to inoculation with the *Brassica rapa* derived isolates from Willamette Valley, Oregon, USA.

Cultivar/Line	*Brassica* Species	*B. rapa*-Derived Isolates						Inferred Phenotype
		Phl002	Phl003	Phl004	Phl005	Phl006	Phl007	
Westar	*Brassica napus*	4.50 ^a,b^	4.40	4.57	5.29	4.83	4.29	IR ^c^
Jet Neuf	*Brassica napus*	3.00	4.33	4.86	4.86	3.29	4.14	R
Surpass 400	*Brassica napus*	3.71	3.57	2.69	2.43	2.71	2.71	R
01-23-2-1	*Brassica napus*	5.33	4.86	3.83	3.43	2.83	2.57	R
Quinta	*Brassica napus*	3.67	5.00	3.00	2.60	2.33	2.79	R
1065	*Brassica napus*	4.50	6.33	5.33	4.57	4.00	4.67	IR
Glacier	*Brassica napus*	3.00	3.80	3.60	2.25	2.60	3.20	R
1135	*Brassica napus*	2.14	2.29	1.57	1.57	2.14	1.57	R
Goéland	*Brassica napus*	1.50	2.67	2.40	1.71	1.10	1.50	R
02-22-2-1	*Brassica napus*	4.17	4.29	4.43	4.43	4.86	4.71	R
Varox	*Brassica juncea*	3.00	2.67	3.00	3.83	2.67	3.00	R
Estilin	*Brassica juncea*	2.67	3.83	3.83	3.83	2.17	1.83	R
UM3309	*Brassica juncea*	3.00	2.33	1.67	2.67	1.83	2.67	R
Forge	*Brassica juncea*	5.17	6.17	6.17	7.33	6.17	6.50	S
Vox-0	*Brassica juncea*	4.57	2.67	3.00	3.83	2.67	3.00	R
Dohirda	*Brassica juncea*	3.83	3.00	2.33	1.83	2.67	2.67	R
CBM	*Brassica juncea*	6.17	7.00	6.17	6.17	6.83	6.67	S
UM1112	*Brassica rapa*	6.67	6.34	6.34	6.67	6.83	6.17	S
UM1161	*Brassica rapa*	7.33	6.17	6.17	6.50	6.34	6.67	S
UM1402	*Brassica rapa*	2.67	3.83	2.33	3.00	2.67	2.67	R
UM1113	*Brassica rapa*	6.17	6.34	6.67	7.33	6.00	5.67	S
UM1147	*Brassica rapa*	2.67	3.00	1.83	2.33	2.33	2.67	R
UM1154	*Brassica rapa*	3.00	3.00	2.67	1.83	2.67	2.33	R

^a^ Score averaged for six plants. ^b^ Pathogenicity assessments completed 11 days’ post-inoculation(dpi). ^c^ R, IR, S = *Brassica* spp. or cultivars that displayed resistance, intermediate resistance, and susceptibility, respectively, to *B. rapa*-derived *Leptosphaeria* isolates from Oregon, USA.

**Table 2:** Sequences of primer pairs used in PCR assays to confirm the identity of *Leptosphaeria biglobosa* and *L. maculans* isolates in this study.

Primer Name ^a^	Sequence (5′–3′)	Annealing Temperature (°C)	Reference
ITS F (PN3)	CCGTTGGTGAACCAGCGGAGGGATC	58	Mendes-Pereira et al. (2003) [6]
ITS R (PN10)	TCCGCTTATTGATATGCTTAAG		
Actin F	GAGCAGGAGATCCAGACTGC	56	Van de Wouw et al. (2008) [10]
Actin R	TTCGAGATCCACATCTGCTG		
β-tubulin F	GTCGAGAACTCCGACGAGAC	55	Van de Wouw et al. (2008) [10]
β-tubulin R	ATCTGGTCCTCGACCTCCTT		
MAT1.1-F	CTCGATGCAATGTACTTGG	56	Cozijinsen and Howlett (2003) [27]
MAT1.2-F	AGCCGGAGGTGAAGTTGAAGCCG		
MAT-R	TGGCGAATTAAGGGATTGCTG		

## Supplementary Materials

Supplementary materials can be found at https://www.mdpi.com/1422-0067/20/7/1668/s1. Table S1. List and characteristics of *Leptosphaeria* isolates used in this study for phylogenetic and/or pathogenicity analyses. Table S2. *Brassica* species and cultivars/lines used to test the pathogenicity of *B. rapa*-derived isolates of *Leptosphaeria biglobosa* from the Willamette Valley of Oregon, USA. Table S3. Sequence similarity analysis of conserved DNA regions of *Leptosphaeria* isolates obtained from *Brassica* spp. Figure S1. Nucleotide sequence alignment of the internal transcribed spacer (ITS) region of ribosomal DNA (rDNA) for isolates of different subclades of *Leptosphaeria biglobosa*. The level of shading corresponds to the level of conservation of the residues. The asterisks represent the boundary of every 20 nucleotides. Sequence data of *L. biglobosa* ‘erysimii’ (isolate AJ550852) is from the NCBI database. *L. maculans* ‘lepidii’ (IBCN84) and *L. maculans* ‘brassicae’ were used as an outgroup. The Brassica rapa derived isolates from a certified organic vegetable seed crop in the Willamette Valley of Oregon were characterized and named as isolates of a new subclade, *L. biglobosa* ‘americensis’. Figure S2. Nucleotide sequence alignment of the *actin* region of DNA of isolates of different subclades of *Leptosphaeria biglobosa*. The level of shading corresponds to the level of conservation of the residues. The asterisks represent the boundary of every 20 nucleotides. Sequence data of *L. biglobosa* ‘erysimii’ (isolate AY748960), *L. biglobosa* ‘australensis’ (AY748952), *L. biglobosa* ‘brassicae’ (AY748949), and *L. biglobosa* ‘thlaspii’ (AY748961) are from the NCBI database. *L. maculans* ‘brassicae’ (Phl010) was used as an outgroup. The *Brassica rapa* derived isolates from a certified organic vegetable seed crop in the Willamette Valley of Oregon were characterized and named as isolates of a new subclade, *L. biglobosa* ‘americensis’. Figure S3. Nucleotide sequence alignment of the *β-tubulin* region of DNA for isolates of different subclades of *Leptosphaeria biglobosa*. The level of shading corresponds to the level of conservation of the residues. The asterisks represent the boundary of every 20 nucleotides. Sequence data of *L. biglobosa* ‘erysimii’ (isolate AY749008), *L. biglobosa* ‘australensis’ (AY749001), *L. biglobosa* ‘brassicae’ (AY748997), and *L. biglobosa* ‘thlaspii’ (AY749009) are from the NCBI database. *L. maculans* ‘brassicae’ (Phl010) was used as an outgroup. The *Brassica. rapa* derived isolates from a certified organic vegetable seed crop in the Willamette Valley of Oregon were characterized and named as isolates of a new subclade, *L. biglobosa* ‘americensis’. Figure S4. Phylogenetic analysis of the *Leptosphaeria maculans*–*L*. *biglobosa* species complex based on neighbor-joining analysis of the sequence of the internal transcribed spacer (ITS) region of ribosomal DNA. The tree was similar to trees constructed by maximum parsimony (MP) or maximum likelihood (ML) analyses. Three *L. maculans* isolates with asterisks were included as outgroup control isolates. The reference sequences of *L. biglobosa* subspecies derived from the NCBI database are each noted with an asterisk and include isolates of *L. biglobosa* ‘canadensis’, *L. biglobosa* ‘occiaustralensis’, *L. biglobosa* ‘australensis’, *L. biglobosa* ‘brassicae’, *L. biglobosa* ‘thlaspii’, and *L. biglobosa* ‘erysimii’ as described in Table S1. Figure S5. Phylogenetic analysis of the *Leptosphaeria maculans*–*L*. *biglobosa* species complex based on neighbor-joining analysis for the *actin* gene sequence. The tree was similar to trees constructed by the maximum parsimony (MP) or maximum likelihood (ML) methods. Two *L. maculans* isolates with asterisks were included as outgroup control isolates. The reference sequences of *L. biglobosa* subspecies derived from NCBI are each noted with an asterisk, including isolates of *L. biglobosa* ‘canadensis’, *L. biglobosa* ‘occiaustralensis’, *L. biglobosa* ‘australensis’, *L. biglobosa* ‘brassicae’, *L. biglobosa* ‘thlaspii’, and *L. biglobosa* ‘erysimii’ as described in Table S1. Figure S6. Phylogenetic analysis of the *Leptosphaeria maculans*–*L*. *biglobosa* species complex based on the neighbor-joining analysis of the *β-tubulin* gene sequence. The tree was similar to trees constructed by maximum parsimony (MP) or maximum likelihood (ML) methods. Two *L. maculans* isolates with asterisks were included as outgroup control isolates. The reference sequences of *L. biglobosa* subspecies derived from NCBI are each noted with an asterisk, including isolates of *L. biglobosa* ‘canadensis’, *L. biglobosa* ‘occiaustralensis’, *L. biglobosa* ‘australensis’, *L. biglobosa* ‘brassicae’, *L. biglobosa* ‘thlaspii’, and *L. biglobosa* ‘erysimii’ as described in Table S1. Figure S7. Disease symptoms on cotyledons of *Brassica rapa* cv., Mizspoona (a); *B. oleracea* cabbage cv., Copenhagen Market (b); *Brassica juncea* cv., Forge (c); and *B. juncea* cv., common brown mustard (CBM) (d) 14 days’ post-inoculation with water, *Leptosphaeria biglobosa* (Lb) ‘americensis’ isolate Phl004, *L. biglobosa* (Lb) ‘brassicae’ isolate LL1-PG1, *L. biglobosa* (Lb) 06J37, *L. biglobosa* (Lb) ‘occiaustralensis’ isolate 14P207, and *L. maculans* isolate 06LM (left to right, respectively).
