# Supplementary material for: A New Subclade of Leptosphaeria biglobosa Identified from Brassica rapa
Source: Int J Mol Sci. 2019 Apr 3;20(7):1668. doi: 10.3390/ijms20071668 (PMC6479289; doi:10.3390/ijms20071668)
Supplement: Supplementary file 1 [file ijms-20-01668-s001.pdf]

Table S1 List and characteristics of *Leptosphaeria* isolates used in this study for phylogenetic and/or pathogenicity analyses.

Isolates ID	Species	Isolated from		ITS size[a]	Sequencing			Phylogenetic/pathogenicity analysis			
		Host Plant	Origin		ITS	*actin*	*β -tubulin*	ITS	*actin*	*β -tubulin*	Concatenated
Phl002	*L. biglobosa*	*Brassica rapa* seed crop	Willamette Valley, Oregon, US	580-585 bp	+[b]	+	+	Included[e]	Included	Included	Included
Phl003	*L. biglobosa*	*Brassica rapa* seed crop	Willamette Valley, Oregon, US	580-585 bp	+	+	+	Included	Included	Included	Included
Phl004	*L. biglobosa*	*Brassica rapa* seed crop	Willamette Valley, Oregon, US	580-585 bp	+	+	+	Included	Included	Included	Included
Phl005	*L. biglobosa*	*Brassica rapa* seed crop	Willamette Valley, Oregon, US	580-585 bp	+	+	+	Included	Included	Included	Included
Phl006	*L. biglobosa*	*Brassica rapa* seed crop	Willamette Valley, Oregon, US	580-585 bp	+	+	+	Included	Included	Included	Included
Phl007	*L. biglobosa*	*Brassica rapa* seed crop	Willamette Valley, Oregon, US	580-585 bp	+	+	+	Included	Included	Included	Included
Phl010	*L. maculans*	*Brassica napus* crop	Lewiston, Idaho, US	555-560 bp	+	+	+	Included	Included	Included	Included
Phl011	*L. maculans*	*Brassica napus* crop	Lewiston, Idaho, US	555-560 bp	+	+	+	Included	Included	Included	Included
Phl012	*L. maculans*	*Brassica napus* crop	Lewiston, Idaho, US	555-560 bp	+	+	+	Ni	Ni	Ni	Ni
Phl013	*L. maculans*	*Brassica napus* crop	Lewiston, Idaho, US	555-560 bp	+	+	+	Ni	Ni	Ni	Ni
Phl014	*L. maculans*	*Brassica napus* crop	Lewiston, Idaho, US	555-560 bp	+	+	+	Ni	Ni	Ni	Ni
Phl015	*L. maculans*	*Brassica napus* crop	Lewiston, Idaho, US	555-560 bp	+	+	+	Ni	Ni	Ni	Ni
06J037	*L. biglobosa* ′canadensis′	*Brassica napus* crop	Australia	580-585 bp	+	+	+	Included	Included	Ni	Included
06J043	*L. biglobosa* ′canadensis′	*Brassica napus* crop	Australia	580-585 bp	Nd	Nd	Nd	Ni	Ni	Ni	Ni
06J154	*L. biglobosa* ′canadensis′	*Brassica napus* crop	Australia	580-585 bp	+	+	+	Included	Included	Ni	Included
06J167	*L. biglobosa* ′canadensis′	*Brassica napus* crop	Australia	580-585 bp	Nd	Nd	Nd	Ni	Ni	Ni	Ni
14P090	*L. biglobosa* ′canadensis′	*Brassica napus* crop	Australia	580-585 bp	Nd	Nd	Nd	Ni	Ni	Ni	Ni
14P091	*L. biglobosa* ′canadensis′	*Brassica napus* crop	Australia	580-585 bp	Nd	+	Nd	Ni	Ni	Ni	Ni
14P093	*L. biglobosa* ′canadensis′	*Brassica napus* crop	Australia	580-585 bp	Nd	+	Nd	Ni	Ni	Ni	Ni
14P094	*L. biglobosa* ′canadensis′	*Brassica napus* crop	Australia	580-585 bp	Nd	+	Nd	Ni	Ni	Ni	Ni
06J041	*L. biglobosa* ′canadensis′	*Brassica napus* crop	Australia	580-585 bp	+	+	+	Included	Included	Ni	Included
06J042	*L. biglobosa* ′canadensis′	*Brassica napus* crop	Australia	580-585 bp	+	+	+	Included	Included	Included	Included
06J044	*L. biglobosa* ′canadensis′	*Brassica napus* crop	Australia	580-585 bp	+	+	+	Included	Included	Ni	Included
06J045	*L. biglobosa* ′canadensis′	*Brassica napus* crop	Australia	580-585 bp	+	+	+	Included	Included	Included	Included
14P204	*L. biglobosa* ′occiaustralensis′	*Brassica napus* crop	Australia	580-585 bp	+	+	+	Included	Ni	Included	Included
14P205	*L. biglobosa* ′occiaustralensis′	*Brassica napus* crop	Australia	580-585 bp	+	+	+	Included	Ni	Ni	Included
14P206	*L. biglobosa* ′occiaustralensis′	*Brassica napus* crop	Australia	580-585 bp	+	+	+	Included	Included	Included	Included
14P207	*L. biglobosa* ′occiaustralensis′	*Brassica napus* crop	Australia	580-585 bp	+	+	+	Included	Ni	Ni	Included
14P208	*L. biglobosa* ′occiaustralensis′	*Brassica napus* crop	Australia	580-585 bp	Nd	Nd	Nd	Ni	Ni	Ni	Ni
Mu7	*L. biglobosa* ′occiaustralensis′	*Brassica napus* crop	Australia	580-585 bp	+	+	+	Included	Included	Included	Included
IBCN65	*L. biglobosa* 'thlaspii'	Na	Canada	580-585 bp	+	+	+	Included	Ni	Ni	Included
IBCN84	*L. maculans* 'lepidii'	Na	Canada	555-560 bp	+	+	+	Included	Ni	Ni	Ni
IBCN89	L. biglobosa 'brassicae'	Na	Canada	580-585 bp	+	+	+	Included	Ni	Ni	Included
IBCN91	L. biglobosa 'australensis'	Na	Australia	580-585 bp	+	+	+	Included	Ni	Ni	Included
61-2	*L. biglobosa* ‘canadensis'	*Brassica napus* crop	Manitoba, 2012	580-585 bp	+	+	+	Ni	Ni	Ni	Ni
151-1	*L. biglobosa* ‘canadensis'	*Brassica napus* crop	Manitoba, 2013	580-585 bp	+	+	+	Ni	Ni	Ni	Ni
PC2-13	*L. biglobosa* ‘canadensis'	*Brassica napus* crop	Na	580-585 bp	+	+	+	Ni	Ni	Ni	Ni
SW4-10-1	*L. biglobosa* ‘canadensis'	*Brassica napus* crop	Na	580-585 bp	+	+	+	Ni	Ni	Ni	Ni
SF-11-7	*L. biglobosa* ‘canadensis'	*Brassica napus* crop	Na	580-585 bp	+	+	+	Ni	Ni	Ni	Ni

SB-9-3	*L. biglobosa* 'canadensis'	*Brassica napus* crop	Na	580-585 bp	+	+	+	Ni	Ni	Ni	Ni
Gr8-17-9	*L. biglobosa* 'canadensis'	*Brassica napus* crop	Na	580-585 bp	+	+	+	Ni	Ni	Ni	Ni
SL4-15-8	*L. biglobosa* 'canadensis'	*Brassica napus* crop	Na	580-585 bp	+	+	+	Ni	Ni	Ni	Ni
SB-6-2	*L. biglobosa* 'canadensis'	*Brassica napus* crop	Na	580-585 bp	+	+	+	Ni	Ni	Ni	Ni
SK12-11-1	*L. biglobosa* 'canadensis'	*Brassica napus* crop	Na	580-585 bp	+	+	+	Ni	Ni	Ni	Ni
SF8-20-3	*L. biglobosa* 'canadensis'	*Brassica napus* crop	Na	580-585 bp	+	+	+	Ni	Ni	Ni	Ni
Br5-11	*L. biglobosa* 'canadensis'	*Brassica napus* crop	Manitoba, 2012	580-585 bp	+	+	+	Ni	Ni	Ni	Ni
Br7-6	*L. biglobosa* 'canadensis'	*Brassica napus* crop	Manitoba, 2012	580-585 bp	+	+	+	Ni	Ni	Ni	Ni
Br7-7	*L. biglobosa* 'canadensis'	*Brassica napus* crop	Manitoba, 2012	580-585 bp	+	+	+	Ni	Ni	Ni	Ni
Br7-13	*L. biglobosa* 'canadensis'	*Brassica napus* crop	Manitoba, 2012	580-585 bp	+	+	+	Ni	Ni	Ni	Ni
Br10-8	*L. biglobosa* 'canadensis'	*Brassica napus* crop	Manitoba, 2012	580-585 bp	+	+	+	Ni	Ni	Ni	Ni
Br10-10	*L. biglobosa* 'canadensis'	*Brassica napus* crop	Manitoba, 2012	580-585 bp	+	+	+	Ni	Ni	Ni	Ni
Br10-11	*L. biglobosa* 'canadensis'	*Brassica napus* crop	Manitoba, 2012	580-585 bp	+	+	+	Ni	Ni	Ni	Ni
Br10-15	*L. biglobosa* 'canadensis'	*Brassica napus* crop	Manitoba, 2012	580-585 bp	+	+	+	Ni	Ni	Ni	Ni
Br17-9	*L. biglobosa* 'canadensis'	*Brassica napus* crop	Manitoba, 2012	580-585 bp	+	+	+	Ni	Ni	Ni	Ni
Br20-6	*L. biglobosa* 'canadensis'	*Brassica napus* crop	Manitoba, 2012	580-585 bp	+	+	+	Ni	Ni	Ni	Ni
MD6-10	*L. biglobosa* 'canadensis'	*Brassica napus* crop	Manitoba, 2012	580-585 bp	+	+	+	Ni	Ni	Included	Included
MD7-8	*L. biglobosa* 'canadensis'	*Brassica napus* crop	Manitoba, 2012	580-585 bp	+	+	+	Ni	Ni	Ni	Ni
MD15-15	*L. biglobosa* 'canadensis'	*Brassica napus* crop	Manitoba, 2012	580-585 bp	+	+	+	Ni	Ni	Ni	Ni
K17	*L. biglobosa* 'canadensis'	Dockage of canola seeds	Na	580-585 bp	+	+	+	Ni	Ni	Ni	Ni
K19	*L. biglobosa* 'canadensis'	Dockage of canola seeds	Na	580-585 bp	+	+	+	Ni	Ni	Ni	Ni
K20	*L. biglobosa* 'canadensis'	Dockage of canola seeds	Na	580-585 bp	+	+	+	Ni	Ni	Ni	Ni
M1	*L. biglobosa* 'canadensis'	Dockage of canola seeds	Na	580-585 bp	+	+	+	Ni	Ni	Ni	Ni
M2	*L. biglobosa* 'canadensis'	Dockage of canola seeds	Na	580-585 bp	+	+	+	Ni	Ni	Ni	Ni
Q1	*L. biglobosa* 'canadensis'	Dockage of canola seeds	Na	580-585 bp	+	+	+	Ni	Ni	Ni	Ni
Q2	*L. biglobosa* 'canadensis'	Dockage of canola seeds	Na	580-585 bp	+	+	+	Ni	Ni	Ni	Ni
T10	*L. biglobosa* 'canadensis'	Dockage of canola seeds	Na	580-585 bp	+	+	+	Ni	Ni	Ni	Ni
T12	*L. biglobosa* 'canadensis'	Dockage of canola seeds	Na	580-585 bp	+	+	+	Ni	Ni	Ni	Ni
U95	*L. biglobosa* 'canadensis'	Dockage of canola seeds	Na	580-585 bp	+	+	+	Ni	Ni	Ni	Ni
U96	*L. biglobosa* 'canadensis'	Dockage of canola seeds	Na	580-585 bp	+	+	+	Ni	Ni	Ni	Ni
U97	*L. biglobosa* 'canadensis'	Dockage of canola seeds	Na	580-585 bp	+	+	+	Ni	Ni	Ni	Ni
W33	*L. biglobosa* 'canadensis'	Dockage of canola seeds	Na	580-585 bp	+	+	+	Ni	Ni	Ni	Ni
001-1	*L. biglobosa* 'canadensis'	*Brassica napus* crop	Manitoba, 2014	580-585 bp	+	+	+	Ni	Ni	Ni	Ni
001-2	*L. biglobosa* 'canadensis'	*Brassica napus* crop	Manitoba, 2014	580-585 bp	+	+	+	Ni	Ni	Ni	Ni
001-3	*L. biglobosa* 'canadensis'	*Brassica napus* crop	Manitoba, 2014	580-585 bp	+	+	+	Ni	Ni	Ni	Ni
002-1	*L. biglobosa* 'canadensis'	*Brassica napus* crop	Manitoba, 2014	580-585 bp	+	+	+	Ni	Ni	Ni	Ni
002-2	*L. biglobosa* 'canadensis'	*Brassica napus* crop	Manitoba, 2014	580-585 bp	+	+	+	Ni	Ni	Ni	Ni
003-1	*L. biglobosa* 'canadensis'	*Brassica napus* crop	Manitoba, 2014	580-585 bp	+	+	+	Ni	Ni	Ni	Ni
003-2	*L. biglobosa* 'canadensis'	*Brassica napus* crop	Manitoba, 2014	580-585 bp	+	+	+	Ni	Ni	Ni	Ni
003-3	*L. biglobosa* 'canadensis'	*Brassica napus* crop	Manitoba, 2014	580-585 bp	+	+	+	Ni	Ni	Ni	Ni
005-1	*L. biglobosa* 'canadensis'	*Brassica napus* crop	Manitoba, 2014	580-585 bp	+	+	+	Ni	Ni	Ni	Ni
005-2	*L. biglobosa* 'canadensis'	*Brassica napus* crop	Manitoba, 2014	580-585 bp	+	+	+	Ni	Ni	Ni	Ni
006-2	*L. biglobosa* 'canadensis'	*Brassica napus* crop	Manitoba, 2014	580-585 bp	+	+	+	Ni	Ni	Ni	Ni
007-1	*L. biglobosa* 'canadensis'	*Brassica napus* crop	Manitoba, 2014	580-585 bp	+	+	+	Ni	Ni	Ni	Ni

008-1	*L. biglobosa* 'canadensis'	*Brassica napus* crop	Manitoba, 2014	580-585 bp	+	+	+	Ni	Ni	Ni	Ni
008-2	*L. biglobosa* 'canadensis'	*Brassica napus* crop	Manitoba, 2014	580-585 bp	+	+	+	Ni	Ni	Ni	Ni
008-3	*L. biglobosa* 'canadensis'	*Brassica napus* crop	Manitoba, 2014	580-585 bp	+	+	+	Ni	Ni	Ni	Ni
009-1	*L. biglobosa* 'canadensis'	*Brassica napus* crop	Manitoba, 2014	580-585 bp	+	+	+	Ni	Ni	Ni	Ni
009-2	*L. biglobosa* 'canadensis'	*Brassica napus* crop	Manitoba, 2014	580-585 bp	+	+	+	Ni	Ni	Ni	Ni
010-1	*L. biglobosa* 'canadensis'	*Brassica napus* crop	Manitoba, 2014	580-585 bp	+	+	+	Ni	Ni	Ni	Ni
010-2	*L. biglobosa* 'canadensis'	*Brassica napus* crop	Manitoba, 2014	580-585 bp	+	+	+	Ni	Ni	Ni	Ni
040-2	*L. biglobosa* 'canadensis'	*Brassica napus* crop	Manitoba, 2014	580-585 bp	+	+	+	Ni	Ni	Ni	Ni
040-3	*L. biglobosa* 'canadensis'	*Brassica napus* crop	Manitoba, 2014	580-585 bp	+	+	+	Ni	Ni	Ni	Ni
040-4	*L. biglobosa* 'canadensis'	*Brassica napus* crop	Manitoba, 2014	580-585 bp	+	+	+	Ni	Ni	Ni	Ni
041-4	*L. biglobosa* 'canadensis'	*Brassica napus* crop	Manitoba, 2014	580-585 bp	+	+	+	Ni	Ni	Ni	Ni
041-7	*L. biglobosa* 'canadensis'	*Brassica napus* crop	Manitoba, 2014	580-585 bp	+	+	+	Ni	Ni	Ni	Ni
043-3	*L. biglobosa* 'canadensis'	*Brassica napus* crop	Manitoba, 2014	580-585 bp	+	+	+	Ni	Ni	Ni	Ni
043-4	*L. biglobosa* 'canadensis'	*Brassica napus* crop	Manitoba, 2014	580-585 bp	+	+	+	Ni	Ni	Ni	Ni
043-5	*L. biglobosa* 'canadensis'	*Brassica napus* crop	Manitoba, 2014	580-585 bp	+	+	+	Ni	Ni	Ni	Ni
NW-1	*L. biglobosa* 'canadensis'	*Brassica napus* crop	Manitoba, 2014	580-585 bp	+	+	+	Ni	Ni	Ni	Ni
NW-2	*L. biglobosa* 'canadensis'	*Brassica napus* crop	Manitoba, 2014	580-585 bp	+	+	+	Ni	Ni	Ni	Ni
NW-4	*L. biglobosa* 'canadensis'	*Brassica napus* crop	Manitoba, 2014	580-585 bp	+	+	+	Ni	Ni	Ni	Ni
NW-5	*L. biglobosa* 'canadensis'	*Brassica napus* crop	Manitoba, 2014	580-585 bp	+	+	+	Ni	Ni	Ni	Ni
NW-6	*L. biglobosa* 'canadensis'	*Brassica napus* crop	Manitoba, 2014	580-585 bp	+	+	+	Ni	Ni	Ni	Ni
NW-7.	*L. biglobosa* 'canadensis'	*Brassica napus* crop	Manitoba, 2014	580-585 bp	+	+	+	Ni	Ni	Ni	Ni
NW-8	*L. biglobosa* 'canadensis'	*Brassica napus* crop	Manitoba, 2014	580-585 bp	+	+	+	Ni	Ni	Ni	Ni
110C-A	*L. biglobosa* 'canadensis'	*Brassica napus* crop	Manitoba, 2013	580-585 bp	+	+	+	Ni	Ni	Ni	Ni
108C-A	*L. biglobosa* 'canadensis'	*Brassica napus* crop	Manitoba, 2013	580-585 bp	+	+	+	Ni	Ni	Included	Ni
108C-B	*L. biglobosa* 'canadensis'	*Brassica napus* crop	Manitoba, 2013	580-585 bp	+	+	+	Ni	Ni	Ni	Ni
134S-A	*L. biglobosa* 'canadensis'	*Brassica napus* crop	Manitoba, 2013	580-585 bp	+	+	+	Ni	Ni	Ni	Ni
LL-PG1	*L. biglobosa* 'canadensis'	*Brassica napus* crop	Manitoba, 2010	580-585 bp	+	+	+	Included	Included	Ni	Included
SC2-1	*L. biglobosa* 'canadensis'	Dockage of canola seeds	Na	580-585 bp	+	+	+	Included	Included	Ni	Included
SC2-2	*L. biglobosa* 'canadensis'	Dockage of canola seeds	Na	580-585 bp	+	+	+	Included	Included	Ni	Included
SC2-3	*L. biglobosa* 'canadensis'	Dockage of canola seeds	Na	580-585 bp	+	+	+	Included	Included	Ni	Included
B1	*L. biglobosa* 'canadensis'	Dockage of canola seeds	Na	580-585 bp	+	+	+	Included	Included	Included	Included
F1	*L. biglobosa* 'canadensis'	Dockage of canola seeds	Na	580-585 bp	+	+	+	Included	Included	Included	Included
G1	*L. biglobosa* 'canadensis'	Dockage of canola seeds	Na	580-585 bp	+	+	+	Included	Included	Included	Included
J34	*L. biglobosa* 'canadensis'	Dockage of canola seeds	Na	580-585 bp	+	+	+	Included	Ni	Included	Included
J35	*L. biglobosa* 'canadensis'	Dockage of canola seeds	Na	580-585 bp	+	+	+	Included	Included	Included	Included
Br7-11	*L. maculans*	*Brassica napus* crop	Manitoba, 2011	555-560 bp	+	+	+	Ni	Ni	Ni	Ni
Br17-4	*L. maculans*	*Brassica napus* crop	Manitoba, 2011	555-560 bp	+	+	+	Ni	Ni	Ni	Ni
PG1-12-PG3	*L. maculans*	*Brassica napus* crop	Manitoba, 2011	555-560 bp	+	+	+	Ni	Ni	Ni	Ni
06LM	*L. maculans*	*Brassica napus* crop	Manitoba, 2011	555-560 bp	+	+	+	Ni	Ni	Ni	Ni
IBCN29[f]	*L. biglobosa* 'australensis'	GenBank	Australia	-[c]	AJ550869[d]	-	-	Included	-	-	Ni
IBCN30	*L. biglobosa* 'australensis'	GenBank	Australia	-	AJ550871	-	-	-	-	-	Ni
IBCN91	*L. biglobosa* 'australensis'	GenBank	USA	-	AJ550870	-	-	-	-	-	Ni
Gui2a2	*L. biglobosa* 'brassicae'	GenBank	China	-	AJ550858	-	-	Included	-	-	Ni
Gui2a3	*L. biglobosa* 'brassicae'	GenBank	China	-	AJ550861	-	-	-	-	-	Ni

IBCN38	*L. biglobosa* ‘brassicae’	GenBank	EU	-	AJ550860	-	-	-	-	-	Ni
IBCN89	*L. biglobosa* ‘brassicae’	GenBank	Canada	-	AJ550863	-	-	Included	-	-	Ni
IBCN93	*L. biglobosa* ‘brassicae’	GenBank	USA	-	AJ550857	-	-	-	-	-	Ni
IBCN63	*L. biglobosa* ‘canadensis’	GenBank	Canada	-	AJ550868	-	-	Included	-	-	Ni
INCB81	*L. biglobosa* ‘canadensis’	GenBank	Canada	-	AJ550867	-	-	Included	-	-	Ni
IBCN83	*L. biglobosa* ‘erysimii’	GenBank	Canada	-	AJ550872	-	-	Included	-	-	Ni
CBS303.51	*L. biglobosa* ‘thlaspii’	GenBank	EU	-	AJ550892	-	-	Included	-	-	Ni
IBCN65	*L. biglobosa* ‘thlaspii’	GenBank	Canada	-	AJ550891	-	-	-	-	-	Ni
UWA21-8	*L. biglobosa* ‘occiaustralensis’	GenBank	Australia	-	AM410082	-	-	Included	-	-	Ni
Strain2.1	*L. biglobosa* ‘australensis’	GenBank	Australia	-	-	AY748952	-	-	Included	-	Ni
Strain2379-4	*L. biglobosa* ‘brassicae’	GenBank	Canada	-	-	AY748949	-	-	Included	-	Ni
PHW1270	*L. biglobosa* ‘brassicae’	GenBank	USA	-	-	AY748951	-	-	Included	-	Ni
Strain92-30-1	*L. biglobosa* ‘canadensis’	GenBank	Canada	-	-	AY748956	-	-	Included	-	Ni
Ery-2	*L. biglobosa* ‘erysimii’	GenBank	Canada	-	-	AY748960	-	-	Included	-	Ni
Strain92-01-1	*L. biglobosa* ‘thlaspii’	GenBank	Canada	-	-	AY748961	-	-	Included	-	Ni
Strain92-01-2	*L. biglobosa* ‘thlaspii’	GenBank	EU	-	-	AY748962	-	-	Included	-	Ni
Strain2.1	*L. biglobosa* ‘australensis’	GenBank	Australia	-	-	-	AY749000	-	-	Included	Ni
PHW1268	*L. biglobosa* ‘australensis’	GenBank	USA	-	-	-	AY749001	-	-	Included	Ni
Strain2379-4	*L. biglobosa* ‘brassicae’	GenBank	Canada	-	-	-	AY748997	-	-	Included	Ni
PHW1270	*L. biglobosa* ‘brassicae’	GenBank	USA	-	-	-	AY748999	-	-	Included	Ni
Strain92-30-1	*L. biglobosa* ‘canadensis’	GenBank	Canada	-	-	-	AY749004	-	-	-	Ni
Ery-2	*L. biglobosa* ‘erysimii’	GenBank	Canada	-	-	-	AY749008	-	-	Included	Ni
Strain92-01-1	*L. biglobosa* ‘thlaspii’	GenBank	Canada	-	-	-	AY749009	-	-	Included	Ni

[a] Sizes of the polymerase chain reaction fragments of the ITS rDNA of different suclades of *Leptosphaeria* species: 580 to 585 bp is the length of amplified DNA expected for *L. biglobosa* isolates, and 555 to 560 bp indicates the length of DNA amplified for *L. maculans* isolates.

[b]‘+’ indicates sequences of the amplified fragments were obtained. ‘Nd’ indicates sequence was not available for that isolate. ‘Na’ indicates the geographical information was not available.

[c] ‘-‘means there are no data can be used.

[d] Accession numbers according to Mendes-Pereira et al. (2003)[6], Voigt et al. (2005)[11].

[e]‘included’ means these sequences were introduced for phylogeny analysis. ‘Ni’ indicates sequence was not included in phylogeny analysis.

[f] Isolate ID was retrieved from GenBank and used as references of *L. biglobosa* subspecies.

Table S2. *Brassica* species and cultivars/lines used to test the pathogenicity of *B. rapa*-derived isolates of *Leptosphaeria biglobosa* from the Willamette Valley of Oregon, USA.

Brassica species	Cultivar/line	Host resistance genotype[a]	Reference[b]
B. napus	01-23-2-1	*Rlm7*	Dilmaghani *et al*. 2009[9]
B. napus	Quinta	*Rlm1, Rlm3*	Kutcher *et al*. 2010[32]
B. napus	Surpass 400	*LepR3, RlmS*	Larkan *et al*. 2013[33]
B. napus	1065	*LepR1*	Kutcher *et al*. unpublished
B. napus	Glacier	*Rlm2 Rlm3*	-
B. napus	1135	*LepR2*	Kutcher *et al.* unpublished
B. napus	Jet Neuf	*Rlm4*	Gout et al. 2006[34]
B. napus	*Goéland*	*Rlm9*	Balesdent *et al*. 2006[35]
B. napus	02-22-2-1	*Rlm3*	Gout *et al*. 2006[34]
B. napus	Westar	No resistance gene	Balesdent *et al*. 2002[36]
B. juncea	Varox	Uncharacterized	University of Manitoba seed bank
B. juncea	Estilin	Uncharacterized	University of Manitoba seed bank
B. juncea	UM3309	Uncharacterized	University of Manitoba seed bank
B. juncea	Forge	*Rlm6*	University of Manitoba seed bank
B. juncea	Vox-0	Uncharacterized	University of Manitoba seed bank
B. juncea	Dohirda	Uncharacterized	University of Manitoba seed bank
B. juncea	CBM	Uncharacterized	University of Manitoba seed bank
B. juncea	UM3301	Uncharacterized	University of Manitoba seed bank
B. rapa	UM1112	Uncharacterized	University of Manitoba seed bank
B. rapa	UM1433	Uncharacterized	University of Manitoba seed bank
B. rapa	UM1161	Uncharacterized	University of Manitoba seed bank
B. rapa	UM1402	Uncharacterized	University of Manitoba seed bank

B. rapa	UM1403	Uncharacterized	University of Manitoba seed bank
B. rapa	UM1113	Uncharacterized	University of Manitoba seed bank
B. rapa	UM1147	Uncharacterized	University of Manitoba seed bank
B. rapa	UM1154	Uncharacterized	University of Manitoba seed bank

[a] Uncharacterized means the resistance genotype could not be determined.

[b] List citations here

Table S3. Sequence similarity analysis of conserved DNA regions of *Leptosphaeria* isolates obtained from *Brassica* spp..

DNA region	Isolate	BLAST species identification[a]	Identity (%)[b]	Similarity to *L. maculans* (%)[c]
actin	Phl002	*L. biglobosa* 'brassicae' group strain[d]	99.00	93.57
	Phl003	*L. biglobosa* 'brassicae' group strain[d]	99.02	93.58
	Phl004	*L. biglobosa* 'brassicae' group strain[d]	99.00	93.57
	Phl005	*L. biglobosa* 'brassicae' group strain[d]	99.00	93.57
	Phl006	*L. biglobosa* 'brassicae' group strain[d]	99.00	93.56
	Phl007	*L. biglobosa* 'brassicae' group strain[d]	99.01	93.57
	L. biglobosa isolate	*L. biglobosa* 'canadensis' group strain[d]	99.00	93.00
	L. maculans isolate	*L. maculans* 'brassicae' group strain[d]	99.00	-
β-tubulin	Phl002	*L. biglobosa* 'canadensis' *β* -tubulin[d]	99.01	92.45
	Phl003	*L. biglobosa* 'canadensis' *β* -tubulin[d]	99.00	92.46
	Phl004	*L. biglobosa* 'canadensis' *β* -tubulin[d]	99.00	92.46
	Phl005	*L. biglobosa* 'canadensis' *β* -tubulin[d]	99.02	92.46
	Phl006	*L. biglobosa* 'canadensis' *β* -tubulin[d]	99.00	92.45
	Phl007	*L. biglobosa* 'canadensis' *β* -tubulin[d]	99.00	92.46
	L. biglobosa isolate	*L. biglobosa* 'canadensis' group strain[d]	99.00	92.00
	L. maculans isolate	*L. maculans* 'brassicae' group strain[e]	99.00	-

ITS rDNA	Phl002	*L. biglobosa* ITS[e]	99.00	96.00
	Phl003	*L. biglobosa* ITS[e]	99.01	95.59
	Phl004	*L. biglobosa* ITS[e]	99.00	96.00
	Phl005	*L. biglobosa* ITS[e]	99.00	96.00
	Phl006	*L. biglobosa* ITS[e]	99.01	96.01
	Phl007	*L. biglobosa* ITS[e]	99.00	96.00
	L. biglobosa isolate	*L. biglobosa* ITS[e]	99.00	-
	L. maculans isolate	*L. maculans* JN3 'brassicae' group[f]	99.00	-

[a] BLAST = Basic Local Alignment Search Tool. The greatest score between a DNA sequence and homologues in the database are listed in this table.

[b] The sequences in the NCBI database that had the greatest degree of nucleotide identity to the homologous sequences.

[c] The nucleotide identity of *actin*, *β-tubulin*, and ITS rDNA sequences to homologous regions of *L. maculans* isolates. - = sequence was not available.

[d] See Voigt et al. (2005)[11] (Each of one isolate from *L. biglobosa* 'brassicae, *L. biglobosa* 'canadensis ', and *L. maculans* 'brassicae' were included for sequence *actin* gene sequence identity analysis. One isolate from *L. biglobosa* 'canadensis and one isolate from *L. maculans* 'brassicae' were included for *β-tubulin* sequence identity analysis).

[e] See Grandaubert et al. (2014)[4] (One isolate ITS rDNA sequence was used for sequence identity analysis).

[f] See Rouxel et al. (2011)[21] (*L. maculans* 'brassicae' JN3 was used for ITS rDNA sequence identity analysis).

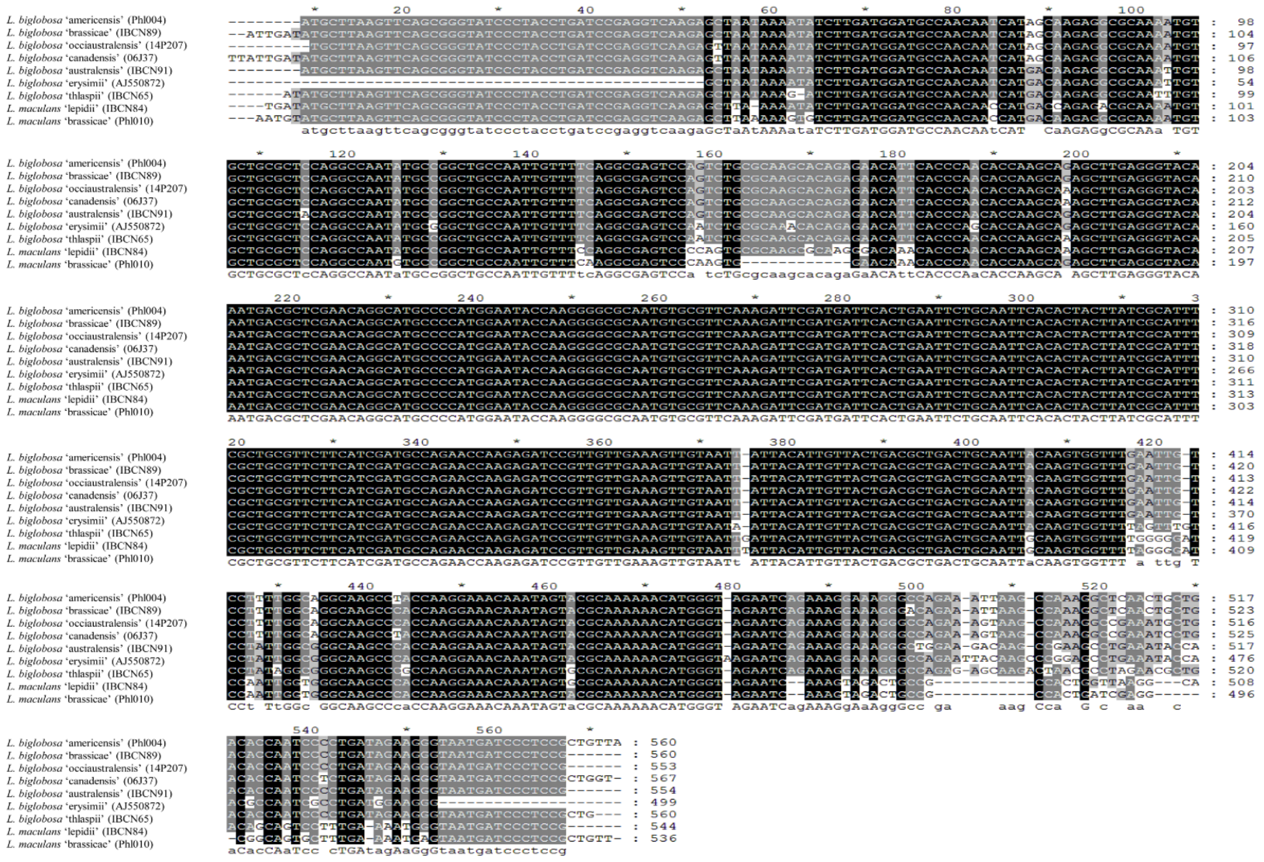

Figure S1. Nucleotide sequence alignment of the internal transcribed spacer (ITS) region of ribosomal DNA (rDNA) for isolates of different subclades of *Leptosphaeria biglobosa*. The level of shading corresponds to the level of conservation of the residues. The asterisks represent the boundary of every 20 nucleotides. Sequence data of *L. biglobosa* 'erysimii' (isolate AJ550852) is from the NCBI database. *L. maculans* 'lepidii' (IBCN84) and *L. maculans* 'brassicae' were used as an outgroup. The *Brassica rapa* derived isolates from a certified organic vegetable seed crop in the Willamette Valley of Oregon were characterized and named as isolates of a new subclade, *L. biglobosa* 'americensis'.

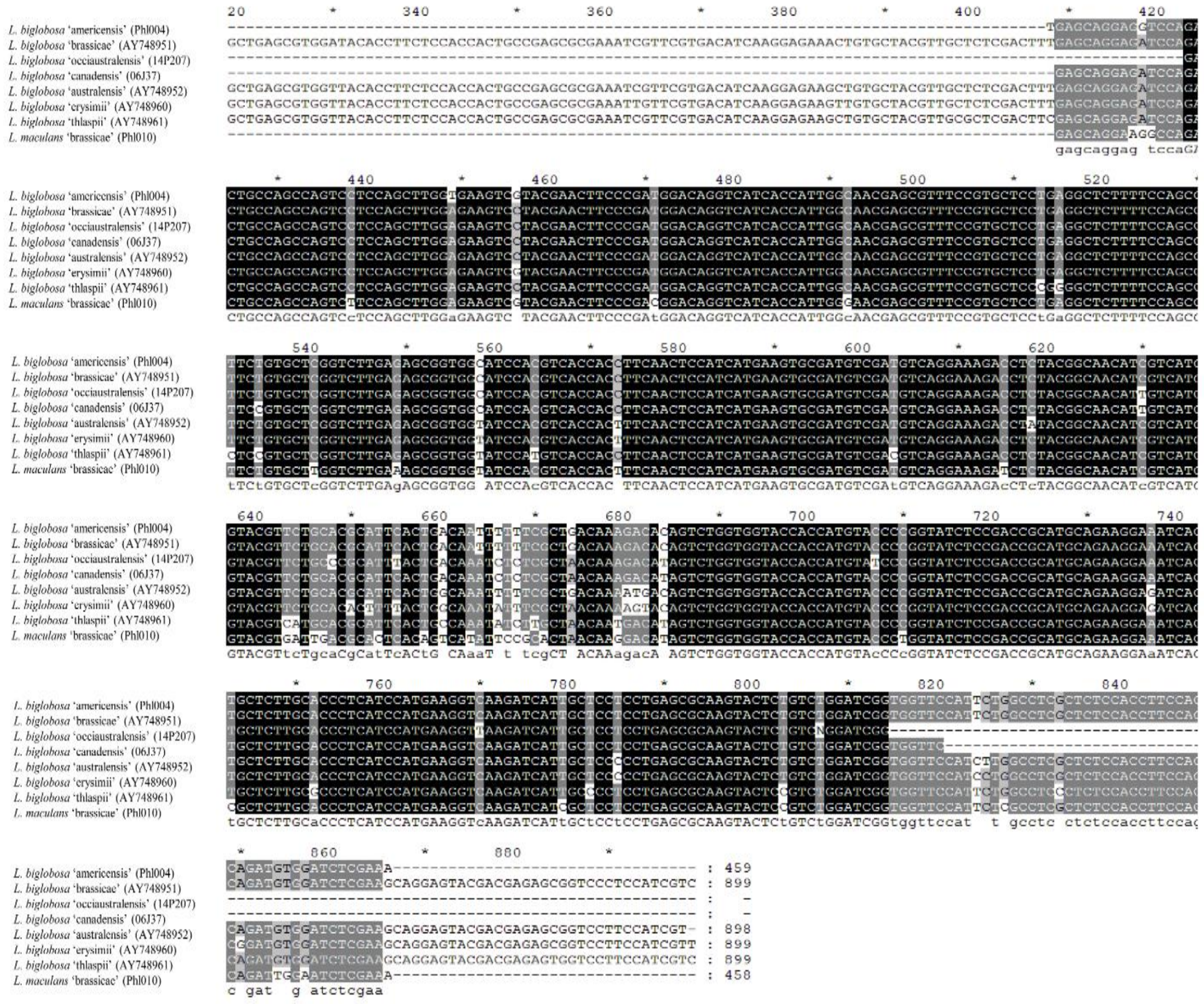

Figure S2. Nucleotide sequence alignment of the *actin* region of DNA of isolates of different subclades of *Leptosphaeria biglobosa*. The level of shading corresponds to the level of conservation of the residues. The asterisks represent the boundary of every 20 nucleotides. Sequence data of *L. biglobosa* 'erysimii' (isolate AY748960), *L. biglobosa* 'australensis' (AY748952), *L. biglobosa* 'brassicae' (AY748949), and *L. biglobosa* 'thlaspii' (AY748961) are from the NCBI database. *L. maculans* 'brassicae' (Phl010) was used as an outgroup. The *Brassica rapa* derived isolates from a certified organic vegetable seed crop in the Willamette Valley of Oregon were characterized and named as isolates of a new subclade, *L. biglobosa* 'americensis'.

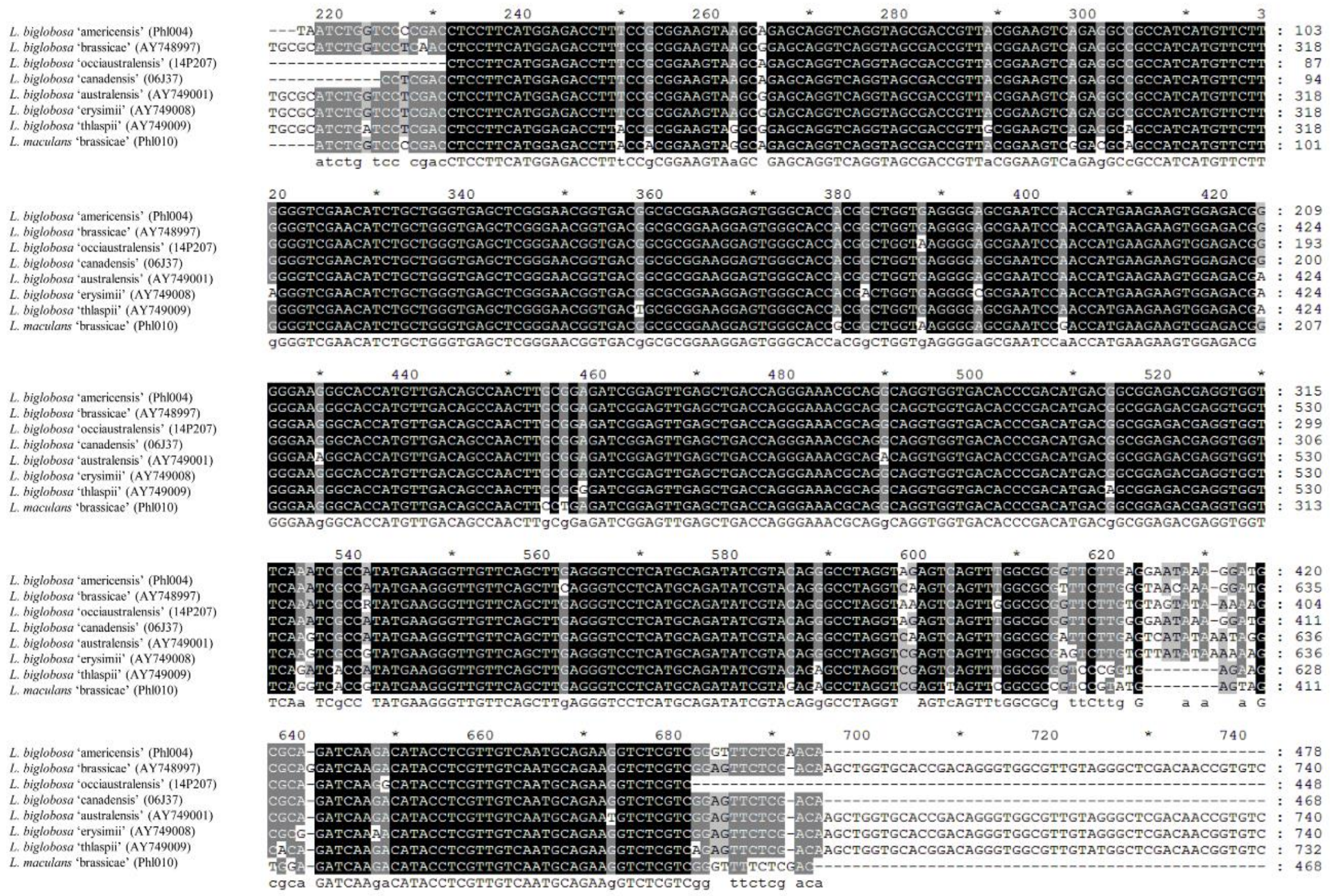

Figure S3. Nucleotide sequence alignment of the *β-tubulin* region of DNA for isolates of different subclades of *Leptosphaeria biglobosa*. The level of shading corresponds to the level of conservation of the residues. The asterisks represent the boundary of every 20 nucleotides. Sequence data of *L. biglobosa* 'erysimii' (isolate AY749008), *L. biglobosa* 'australensis' (AY749001), *L. biglobosa* 'brassicae' (AY748997), and *L. biglobosa* 'thlaspii' (AY749009) are from the NCBI database. *L. maculans* 'brassicae' (Phl010) was used as an outgroup. The *Brassica. rapa* derived isolates from a certified organic vegetable seed crop in the Willamette Valley of Oregon were characterized and named as isolates of a new subclade, *L. biglobosa* 'americensis'.

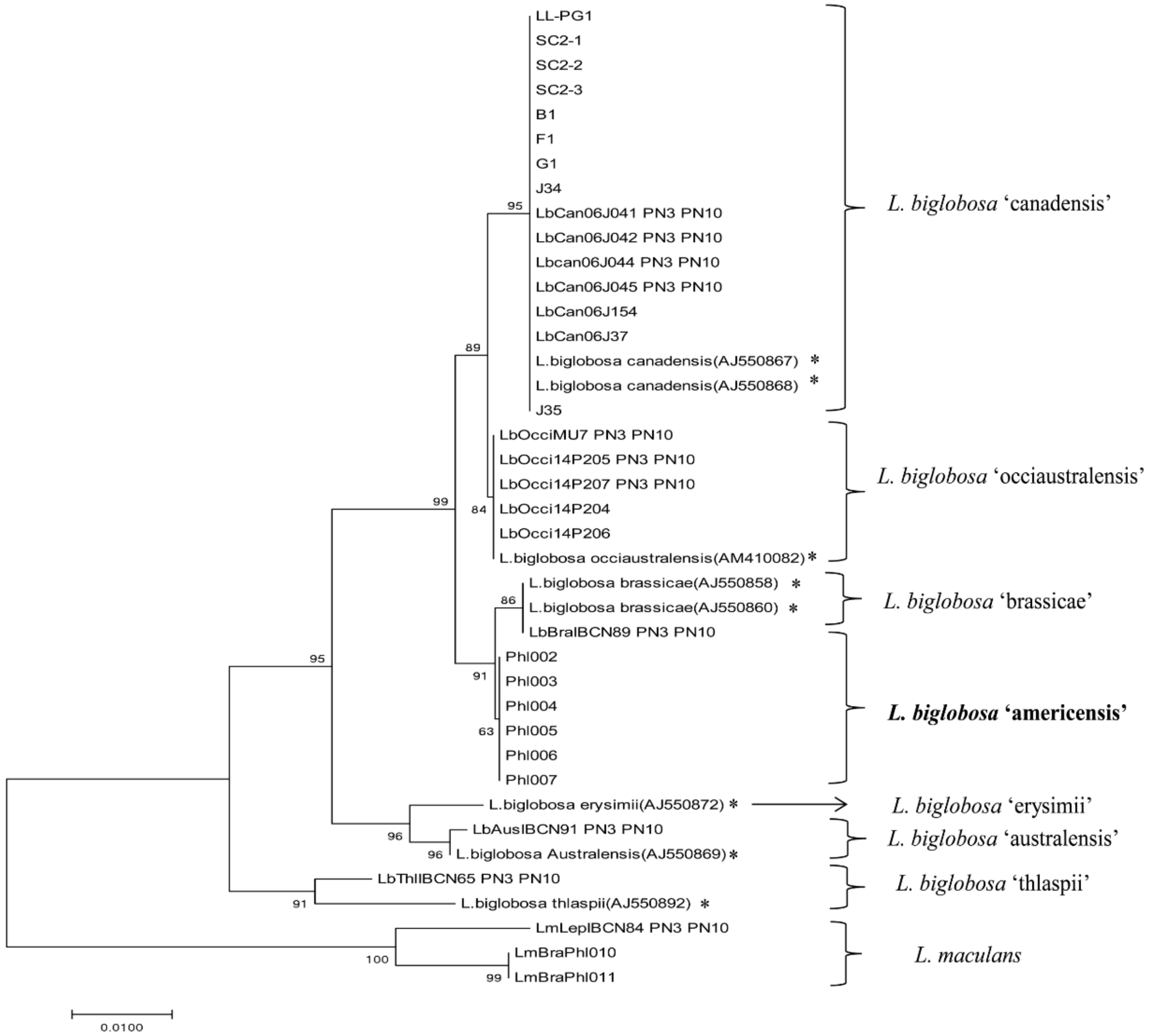

Figure S4. Phylogenetic analysis of the *Leptosphaeria maculans*–*L. biglobosa* species complex based on neighbor-joining analysis of the sequence of the internal transcribed spacer (ITS) region of ribosomal DNA. The tree was similar to trees constructed by maximum parsimony (MP) or maximum likelihood (ML) analyses. Three *L. maculans* isolates with asterisks were included as outgroup control isolates. The reference sequences of *L. biglobosa* subspecies derived from the NCBI database are each noted with an asterisk and include isolates of *L. biglobosa* 'canadensis', *L. biglobosa* 'occiaustralensis', *L. biglobosa* 'australensis', *L. biglobosa* 'brassicae', *L. biglobosa* 'thlaspii', and *L. biglobosa* 'erysimii' as described in Table S1.

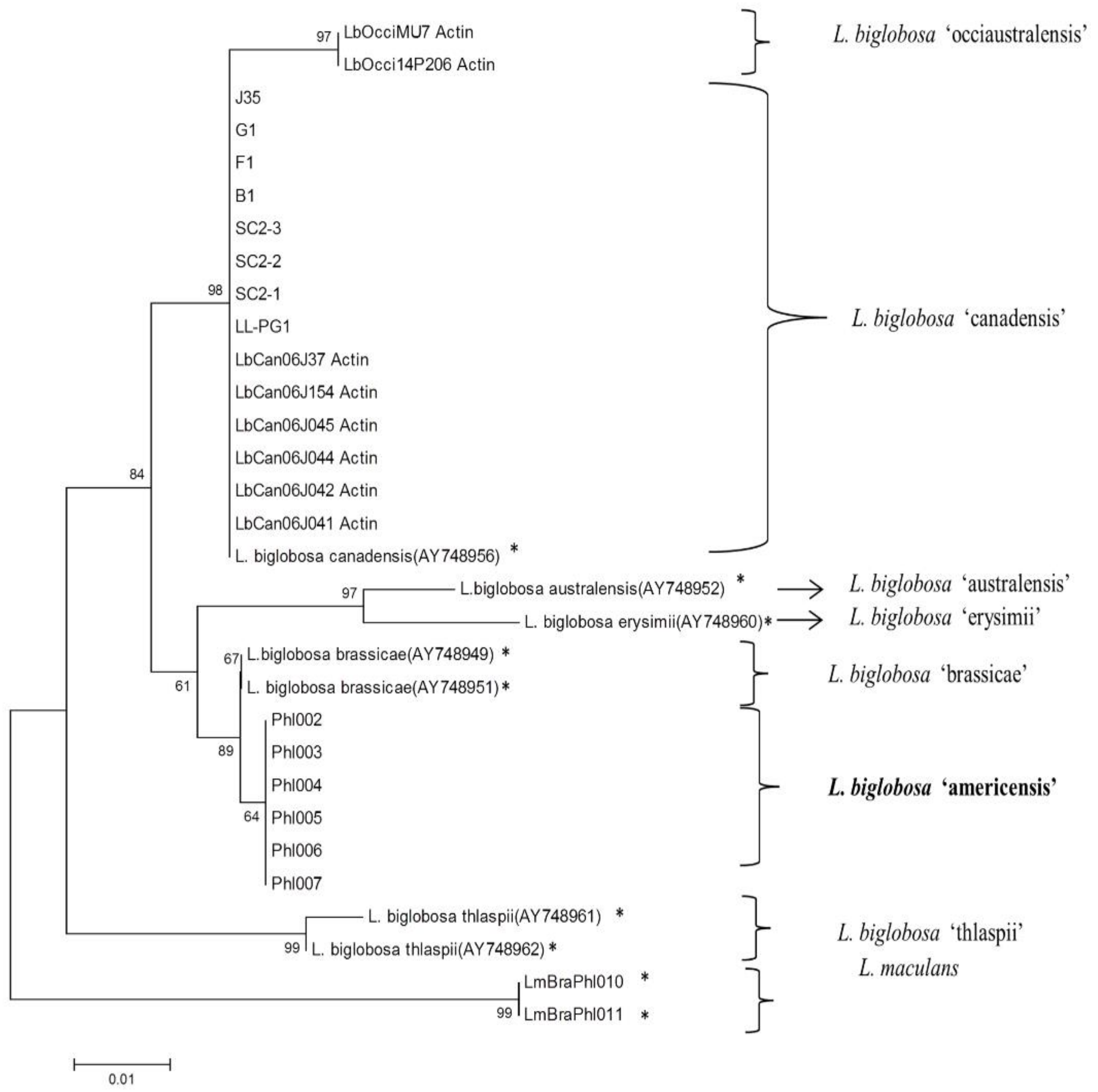

Figure S5. Phylogenetic analysis of the *Leptosphaeria maculans*–*L. biglobosa* species complex based on neighbor-joining analysis for the *actin* gene sequence. The tree was similar to trees constructed by the maximum parsimony (MP) or maximum likelihood (ML) methods. Two *L. maculans* isolates with asterisks were included as outgroup control isolates. The reference sequences of *L. biglobosa* subspecies derived from NCBI are each noted with an asterisk, including isolates of *L. biglobosa* 'canadensis', *L. biglobosa* 'occiaustralensis', *L. biglobosa* 'australensis', *L. biglobosa* 'brassicae', *L. biglobosa* 'thlaspii', and *L. biglobosa* 'erysimii' as described in Table S1.

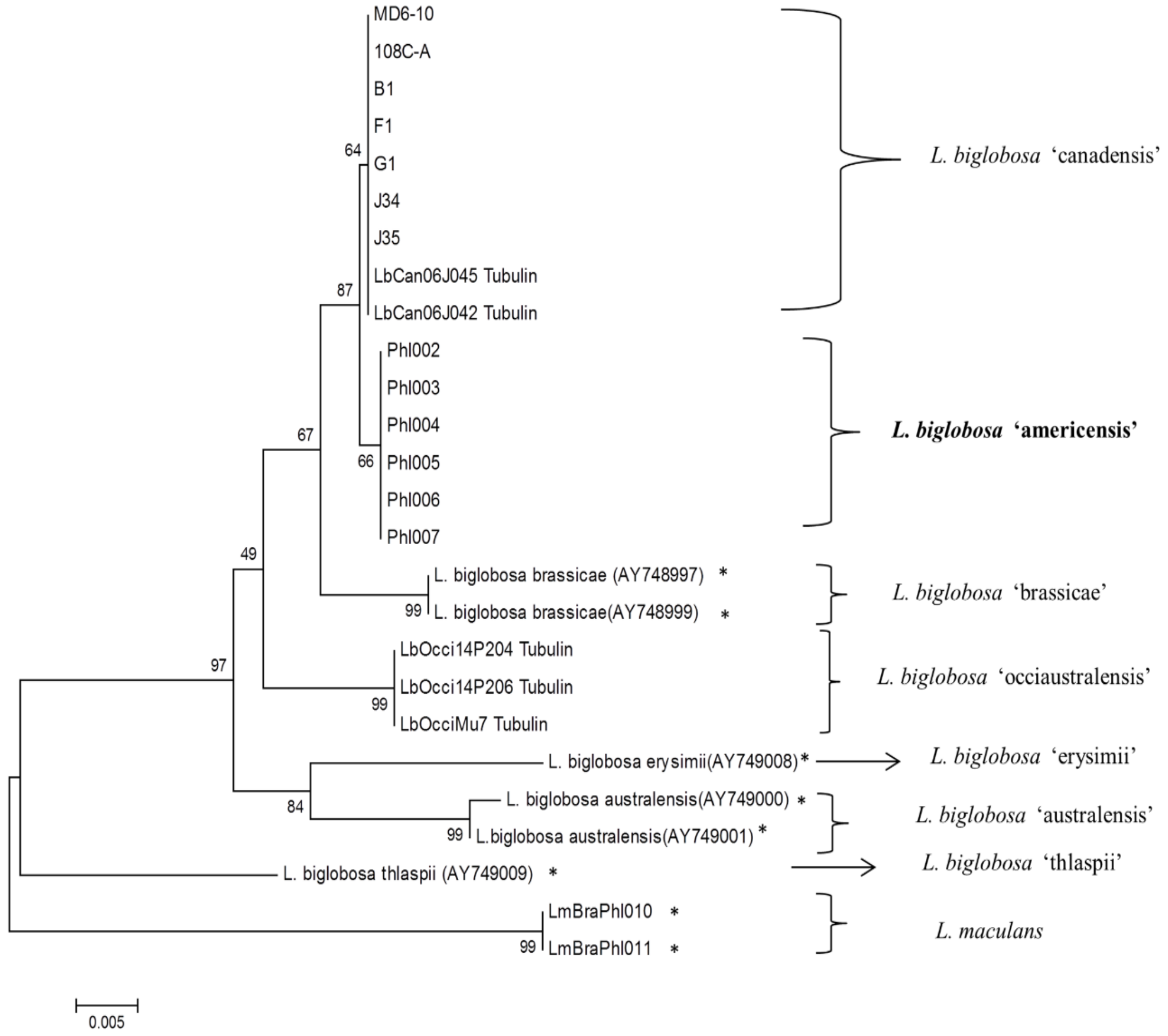

Figure S6. Phylogenetic analysis of the *Leptosphaeria maculans*–*L. biglobosa* species complex based on the neighbor-joining analysis of the *β-tubulin* gene sequence. The tree was similar to trees constructed by maximum parsimony (MP) or maximum likelihood (ML) methods. Two *L. maculans* isolates with asterisks were included as outgroup control isolates. The reference sequences of *L. biglobosa* subspecies derived from NCBI are each noted with an asterisk, including isolates of *L. biglobosa* 'canadensis', *L. biglobosa* 'occiaustralensis', *L. biglobosa* 'australensis', *L. biglobosa* 'brassicae', *L. biglobosa* 'thlaspii', and *L. biglobosa* 'erysimii' as described in Table S1.

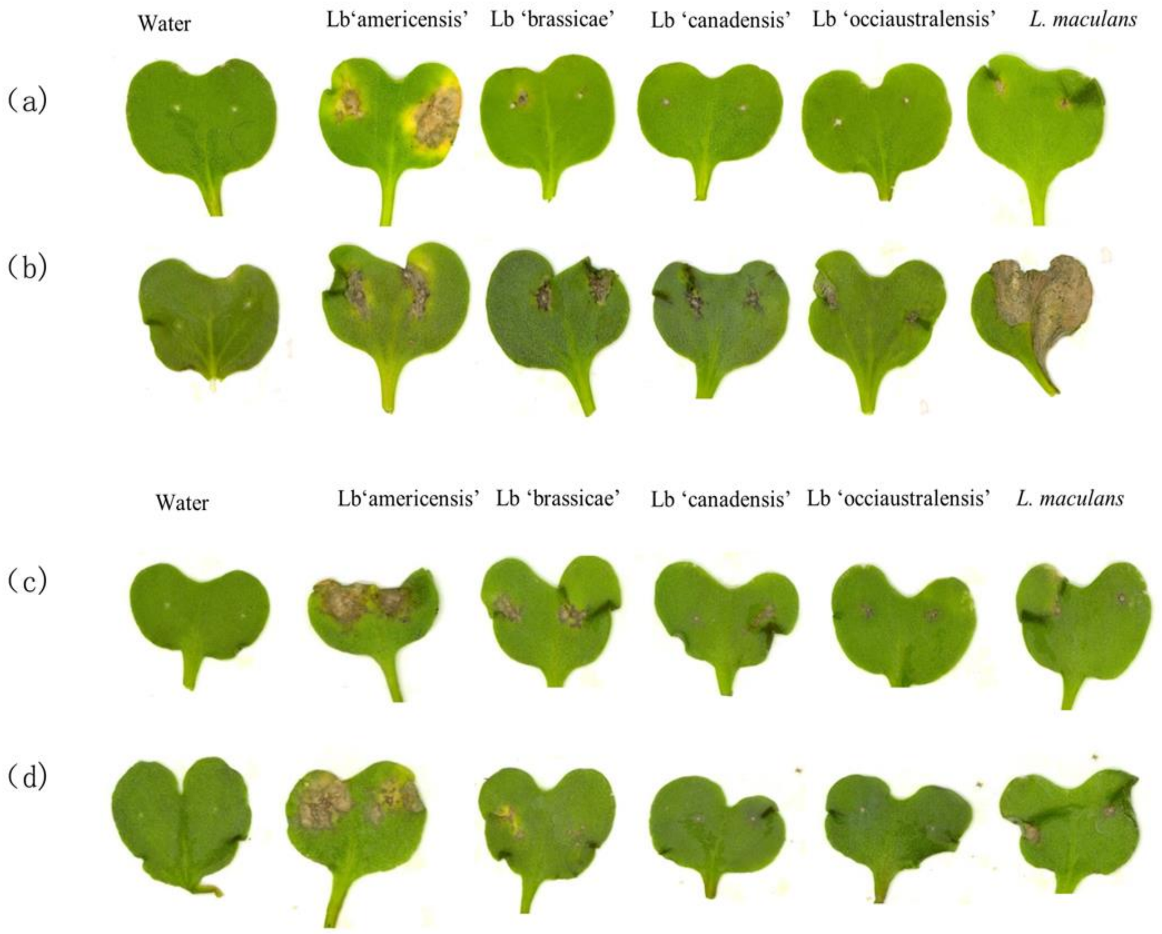

Figure S7. Disease symptoms on cotyledons of *Brassica rapa* cv., Mizspoona (**a**); *B. oleracea* cabbage cv., Copenhagen Market (**b**); *Brassica juncea* cv., Forge (**c**); and *B. juncea* cv., common brown mustard (CBM) (**d**) 14 days' post-inoculation with water, *Leptosphaeria biglobosa* (Lb) 'americensis' isolate Phl004, *L.biglobosa* (Lb) 'brassicae' isolate LL1-PG1, *L.biglobosa* (Lb) 06J37, *L.biglobosa* (Lb) 'occiaustralensis' isolate 14P207, and *L. maculans* isolate 06LM (left to right, respectively).

References

32. Kutcher, H.R.; Yu,F.; Brun, H. Improving blackleg disease management of *Brassica napus* from knowledge of genetic interactions with *Leptosphaeria maculans*. *Can. J. Plant Pathol.* **2010**, *32*, 29–34.

33. Larkan, N.J.; Lydiate, D.J.; Parkin, I.A.P.; Nelson, M.N.; Epp, D.J.; Cowling, W.A.; Rimmer, S.R.; Borhan, M.H. The *Brassica napus* blackleg resistance gene *LepR3* encodes a receptor-like protein triggered by the *Leptosphaeria maculans* effector AVRLM1. *New Phytol.* **2012**, *197*, 595–605.

34. Gout, L.; Fudal, I.; Kuhn, M.L.; Blaise, F.; Eckert, M.; Cattolico, L.; Balesdent, M.H.; Rouxel, T. Lost in the middle of nowhere: The *AvrLm1* avirulence gene of the Dothideomycete *Leptosphaeria maculans*. *Mol. Microbiol.* **2006**, *60*, 67–80.

35. Balesdent, M.H.; Louvard, K.; Pinochet, X.; Rouxel, T. A large-scale survey of races of *Leptosphaeria maculans* occurring on oilseed rape in France. *Eur. J. Plant Pathol*. **2006**, *114*, 53–65.

36. Balesdent, M.H.; Attard, A.; Kuhn, M.L.; Rouxel, T. New avirulence genes in the phytopathogenic fungus *Leptosphaeria maculans*. *Phytopathology* **2002**, *92*, 1122–1133.
